# Park Proximity and Emergency Department Visit Rates for Asthma and Acute Myocardial Infarction in Los Angeles County: A Cross-Sectional Ecological Study

**DOI:** 10.7759/cureus.117415

**Published:** 2026-10-04

**Authors:** Juliana N Fong

**Affiliations:** 1 Urban Studies, Stanford University, Stanford, USA

**Keywords:** air pollution, asthma, ecological study, emergency department utilization, geographic information systems, green space, myocardial infarction, park access, particulate matter, social determinants of health

## Abstract

Background

Parks are promoted as a public health resource, and neighborhoods with limited park access often face co-occurring air pollution and socioeconomic disadvantage. The primary objective of this study was to estimate the association between census tract park proximity and emergency department (ED) visit rates for asthma and acute myocardial infarction (AMI) in Los Angeles County, California, adjusted for fine particulate matter (PM2.5), ozone, and poverty. The two outcomes were co-primary, and no directional hypothesis was prespecified. A secondary objective was to assess whether the association persisted after accounting for spatial dependence.

Methods

This cross-sectional ecological study included 2,305 census tracts with residents and complete data. Park proximity was measured as each tract's mean distance along the 2016 road network of the U.S. Census Bureau (Washington, DC, USA) from its Census 2010 population-weighted center to the three nearest recreational spaces in the 2024 county inventory. The straight-line distance from the tract centroid was used as a sensitivity analysis. Age-adjusted asthma and AMI ED visit rates per 10,000 residents (2015-2017), PM2.5 (2015-2017), ozone (2017-2019), and the percentage of residents below twice the federal poverty level (2015-2019) were drawn from CalEnviroScreen 4.0 (Office of Environmental Health Hazard Assessment, Sacramento, CA, USA). The data years did not fully overlap. Population-weighted regressions with standard errors clustered by ZIP (Zone Improvement Plan) code were compared with spatial error models, and associations were divided into within- and between-ZIP-code components.

Results

In conventional models, an interquartile range (IQR) increase in the park distance (0.38 miles) corresponded to 1.64 more asthma ED visits per 10,000 residents, with a 95% confidence interval (CI) of -0.14 to 3.41 (p = 0.070), and to 0.34 more AMI ED visits (95% CI 0.06 to 0.63; p = 0.018). Park distance explained less than 1% of residual variation. Straight-line distance from the tract centroid gave larger estimates (2.40 and 0.40 per its own IQR of 0.28 miles). Residuals were strongly spatially autocorrelated (Moran's I 0.65 and 0.60). In spatial error models, the park-distance coefficients were negative (asthma -1.01, 95% CI -1.61 to -0.41; AMI -0.12, 95% CI -0.25 to 0.00), and neither was statistically significant after a post hoc exclusion of one peripheral region (-0.46 and -0.09). The positive coefficients arose between ZIP codes rather than within them. In unsmoothed ZIP-code asthma counts, the association was positive when no spatial term was included, but it was absent when one was added. Poverty was positively associated with both outcomes in the main conventional and spatial models and with asthma in the ZIP-code count models.

Conclusions

In conventional models, a greater distance from parks coincided with slightly higher AMI ED visit rates across Los Angeles County, and the asthma estimate was also positive but not statistically significant with the primary street-network measure. Both associations were small, appeared between ZIP codes rather than within them, and did not persist in spatial error models. These ecological data, whose outcomes vary mainly between ZIP codes, cannot separate park-related differences from regional confounding and do not support causal or prescriptive conclusions.

## Introduction

Parks and green spaces are widely promoted as community health resources. Park-based interventions have been associated with improved health-related outcomes in young people [1], and in Los Angeles County, greater neighborhood green space has been associated with longer life expectancy [2]. Asthma, a common chronic respiratory disease, is a particular focus of this literature. Outdoor air pollution is a recognized asthma trigger, and early-life exposure has been associated with the development of childhood asthma [3]. Additionally, asthma-related emergency department (ED) utilization falls disproportionately on low-income communities [4], and prior work in Los Angeles has linked the availability of public parks and open space to lower asthma ED visit rates [5].

Air pollution is one plausible environmental pathway connecting neighborhood conditions to both respiratory and cardiovascular outcomes. Fine particulate matter (PM2.5), particles no larger than 2.5 micrometers in diameter, has been linked to the onset and exacerbation of childhood asthma [6] and to increased respiratory hospitalization [7]. It is also associated with hypertension, myocardial infarction, stroke, and other cardiovascular disease [8], which may reflect the close linkage between the pulmonary and cardiovascular systems [9]. PM2.5 comes both from local sources, such as traffic and residential combustion, and from secondary particles formed in the atmosphere, which reflect regional and long-range transport [10]. In contrast, ozone is not emitted directly but forms from precursor pollutants in sunlight, and near heavy traffic it is depleted by nitric oxide. For example, at a heavily trafficked London roadside site, ozone was lowest in the early morning because of this depletion by nitric oxide (titration), and it was highest in warm, aged air masses [11]. Ozone also contributes to airway inflammation and bronchial hyper-responsiveness [12]. Together, PM2.5 and ozone capture both local and regional sources of air pollution.

Socioeconomic disadvantage is tied to both pollution exposure and health outcomes. Low socioeconomic status is strongly associated with cardiovascular disease [13] and with asthma morbidity and emergency care use, through pathways that include housing quality, healthcare access, and chronic stress [4]. These burdens also overlap with patterns of green space access. At the same time, not all green space is associated with asthma in the same way. In Los Angeles County, tree canopy configuration and small neighborhood green spaces have been associated with fewer asthma ED visits, whereas large parks alone have not [14]. In metropolitan Los Angeles, park-adjacent neighborhoods have been found to have the highest nitrogen dioxide and PM2.5 concentrations, even though the parks themselves had the lowest [15]. Furthermore, environmental injustice, including disproportionate pollution burdens and unequal green space access, persists in greater Los Angeles [16]. Because of this overlap, any apparent association between park proximity and health may partly reflect confounding by pollution and poverty.

Few analyses have examined park access, multiple pollutants, poverty, and more than one health outcome at the neighborhood scale within a single metropolitan area. One difficulty for such analyses is that neighboring areas share environmental and social conditions, so an association estimated across a whole county may reflect broad regional differences rather than local ones. In this study, a tract-level measure of park proximity was constructed from the county's park inventory and a public road network file and joined to publicly available tract-level data on asthma and acute myocardial infarction (AMI) ED visit rates, PM2.5, ozone, and poverty. The years of these data do not fully overlap: the outcomes and PM2.5 cover 2015-2017, ozone 2017-2019, and poverty 2015-2019, while the road network dates from 2016 and the park inventory from 2024. The primary objective was to estimate the cross-sectional ecological association between park proximity, measured along the street network, and age-adjusted asthma and AMI ED visit rates across all Los Angeles County census tracts with residents and complete data, adjusted for PM2.5, ozone, and poverty. The two outcomes were co-primary, and no directional hypothesis was prespecified. Secondary objectives were to describe the county-wide spatial patterning of these variables; to assess whether the association persisted after accounting for spatial dependence, and whether it arose within or between ZIP (Zone Improvement Plan) codes; and to examine its sensitivity to the definition of the sample, population weighting, the standard-error method, the exposure measure, and the timing of the park and ozone data. Analyses labeled post hoc and the additional models reported in the Appendices were exploratory, whereas all other analyses were planned to address these objectives.

## Materials and methods

Study design and setting

This cross-sectional ecological study analyzed census tracts in Los Angeles County, California, and is reported in line with the Strengthening the Reporting of Observational Studies in Epidemiology (STROBE) recommendations for cross-sectional studies [17]. The analysis is descriptive: it estimates tract-level associations and does not test a causal hypothesis. Los Angeles County is the most populous county in the United States and combines a long history of severe air pollution [18] with persistent geographic disparities in pollutant exposure [19], which makes it an informative setting for neighborhood-level environmental health analysis. Health outcome data and PM2.5 cover 2015-2017, while ozone covers 2017-2019 and the poverty measure covers 2015-2019. The park inventory is a 2024 county release compiled from 2023 source datasets, and the road network is the 2016 release.

Units of analysis

The census tract was the unit of analysis. Tracts are small, relatively stable statistical subdivisions designed for describing local populations, and they have a long history of use in public health research [20]. Tract boundaries follow the 2010 census definitions used by the health and pollution data source.

Data sources

Park locations were taken from the Recreational Spaces dataset (Los Angeles County Department of Public Health, Los Angeles, CA, USA), published by the County of Los Angeles (Los Angeles, CA, USA) through its Enterprise geographic information system (GIS) hub as part of the Community Health Profiles Data Initiative [21]. This layer contains 2,509 polygon boundaries for recreational spaces county-wide, ranging from small neighborhood parks to large regional facilities, with attributes including name, acreage, and access type. Its documented inputs include the California Protected Areas Database (GreenInfo Network, Oakland, CA, USA), the Countywide Parks and Open Space dataset (County of Los Angeles, Los Angeles, CA, USA), and the ParkServe dataset (Trust for Public Land, San Francisco, CA, USA). The layer was accessed through ArcGIS Online (Esri, Redlands, CA, USA) in September and October 2025, and the service reports that its data were last edited in January 2024. The road network and the tract population-weighted centers came from the U.S. Census Bureau (Washington, DC, USA): the 2016 TIGER/Line (Topologically Integrated Geographic Encoding and Referencing) All Lines file for the county and the Census 2010 centers of population by tract. All health, pollution, and socioeconomic measures came from CalEnviroScreen 4.0 (Office of Environmental Health Hazard Assessment, Sacramento, CA, USA), the California environmental health screening tool, which compiles tract-level indicators from state and federal sources and reports them on the 2010 tract boundaries used here [22].

Variables

The exposure was park proximity, operationalized as the average of the distances along the street network from a tract's Census 2010 population-weighted center to the nearest boundary of each of the three nearest recreational spaces, in miles. The equivalent walking time at three miles per hour (20 minutes per mile) is reported alongside it. Meanwhile, the straight-line measure, which is the average straight-line distance from the tract's geographic centroid to the three recreational spaces nearest by straight-line distance, is retained as a sensitivity analysis and as the basis of the post hoc peripheral-tract restrictions. For this purpose, a "park" was any feature of the Recreational Spaces layer, without regard to size, amenities, maintenance, or access type. The layer records 2,230 features as open access, 236 as restricted access, and 43 as unknown. Additionally, 478 features overlap another feature because the layer compiles several source datasets. Thus, sensitivity analyses used open-access features only and, separately, a version in which overlapping features were merged. Distances were averaged over the three nearest parks instead of being taken from the single nearest park. This is because a tract whose center happens to sit beside one park would otherwise appear to have excellent park access even where parks are scarce.

The outcomes were the age-adjusted rates of ED visits per 10,000 residents for asthma and for AMI, each averaged over 2015-2017. As documented for CalEnviroScreen 4.0, these rates were first calculated for ZIP codes from statewide ED records and age-adjusted. Rates for ZIP codes with fewer than 12 visits were estimated with a spatial model that combines local and statewide information. ZIP-code rates were then assigned to 2010 census blocks by areal apportionment, and each tract's rate is the population-weighted average of its blocks [22]. Therefore, the outcomes were expected to vary mainly at the scale of ZIP codes, as opposed to individual tracts, and the Results confirm this. AMI was used as the cardiovascular indicator because it is an abrupt, severe event reliably captured in ED data [22].

Covariates, selected a priori as potential confounders, were annual mean PM2.5 concentration (µg/m³, 2015-2017), the mean of summer-month (May-October) daily maximum eight-hour ozone concentrations (parts per million, ppm; 2017-2019), and the percentage of residents living below twice the federal poverty level, from the American Community Survey 2015-2019 estimates (U.S. Census Bureau, Washington, DC, USA). The doubled poverty threshold is the CalEnviroScreen convention that allows for California's high cost of living [22]. The 2017-2019 ozone values were retained as the primary covariate because they are the values released with the outcome data in CalEnviroScreen 4.0 and because tract ozone values in the two periods were highly correlated (0.93). The 2012-2014 values from CalEnviroScreen 3.0 were used in sensitivity analyses. All CalEnviroScreen variables were used as raw indicator values rather than percentiles.

GIS procedures

The straight-line measure was built in ArcGIS Pro (Esri, Redlands, CA, USA) and ArcGIS Online, and the street-network measure was built in R version 4.6.1 (R Foundation for Statistical Computing, Vienna, Austria) and Python 3.14 (Python Software Foundation, Wilmington, DE, USA). For the straight-line measure, Los Angeles County tracts (Federal Information Processing Standards county code 037) were extracted from the CalEnviroScreen 4.0 geodatabase. Tract centroids were generated with the Find Centroids tool, constrained to lie within each tract's boundary. The Find Closest tool (line distance, three closest locations per tract, maximum search distance 30 miles, units in miles) was then used to measure straight-line distances from each centroid to the nearest edge of each of the three nearest park polygons. Every polygon was treated as a separate location, so overlapping features from different source datasets were not merged. Finally, the three distances were averaged per tract with the Summary Statistics tool and joined back to the CalEnviroScreen attributes by tract identifier. Recomputing this measure in R from the same layer and centroids reproduced the recorded values (correlation 0.996; median absolute difference 0.001 miles, although 10 tracts differed by more than 0.25 miles). It was not recorded whether the Find Closest tool measured the line distance geodesically or on a projected plane. However, planar and geodesic recalculations agree with the recorded values equally well. The layer contains no zero-area polygons.

For the street-network measure, the road segments of the 2016 TIGER/Line All Lines file, excluding primary roads (limited-access highways) and ramps, formed an undirected network whose segment lengths were measured in the California Albers projection. The file's other classes, including local streets, private roads, vehicular trails, alleys, and its few pedestrian ways (242 walkway segments), were all treated as walkable. Because limited-access highways and ramps were excluded, a path can cross a freeway only where another road crosses it. Rivers, rail lines, and similar physical barriers are reflected only where they interrupt the road network. Disconnected fragments of fewer than 500 nodes were ignored, which left the main network (99.5% of nodes) and Santa Catalina Island. Park access points were every network node inside a park polygon or within 60 m (about 200 feet) of its boundary, every point where a road segment crosses a park boundary (7,793 points on 968 parks), and the nearest point on the nearest road segment. There were 123 parks with no node within that band, and these were reached through a boundary crossing or the nearest point on the nearest road segment. Each tract's population-weighted center was joined to the nearest point on the nearest road segment (median 21 m, about 70 feet, away). However, 13 centers lay more than 0.25 miles from the network, the farthest 2.8 km, because they lie in mountain tracts or near ignored fragments. A center that itself lay inside a park or within 60 m of its boundary (170 tracts) reached that park directly at the straight-line distance. The straight-line connectors at both ends were added to the shortest path along the network, which was computed with Dijkstra's algorithm. Finally, the three smallest distances to distinct parks were averaged. In the primary sample, network distances were never shorter than the straight-line distances from the same centers (median ratio 1.39; correlation 0.90), and the network measure correlated 0.83 with the straight-line measure from the tract centroid.

Maps were produced on an OpenStreetMap basemap (OpenStreetMap Foundation, Cambridge, United Kingdom) [23] using quantile classification. This method was selected to show relative spatial contrasts, as opposed to absolute thresholds. The study area, its major regions, and the census tract boundaries are shown in Figure 1.

**Figure 1:**
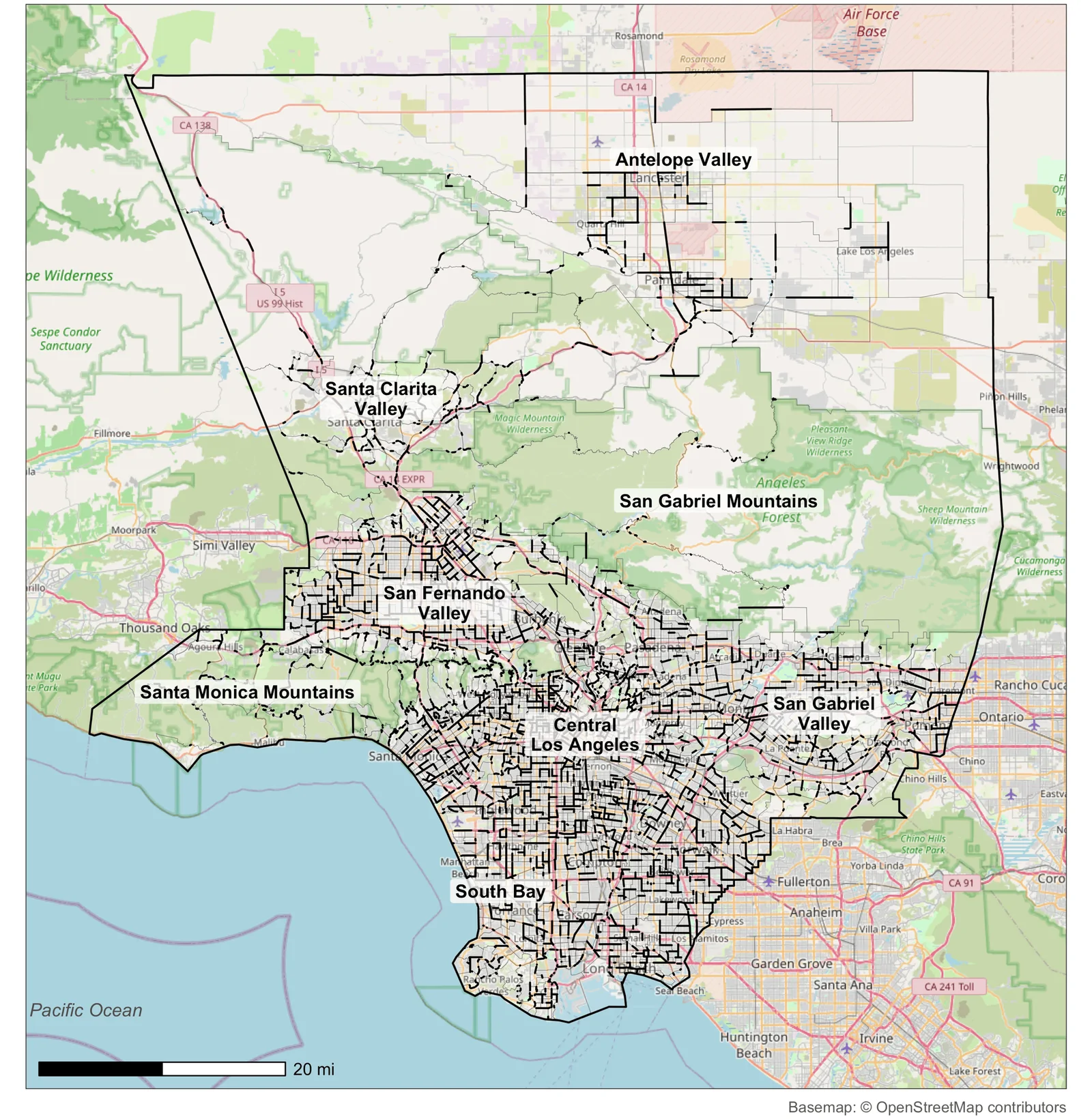
Study area: census tracts and major regions of Los Angeles County, California Boundaries show the 2,343 Los Angeles County census tracts in the CalEnviroScreen 4.0 extract (2010 census definitions). Labels mark the county's major geographic regions, including the Antelope Valley and San Fernando Valley referred to in the Results. The county's two Channel Island tracts lie outside the map frame. Basemap: © OpenStreetMap contributors, available under the Open Database License (https://www.openstreetmap.org/copyright) [23]; reproduced under that license, which requires attribution only.

Study sample

Of the county's 2,346 census tracts (2010 boundaries), 2,343 appeared in the CalEnviroScreen 4.0 release. CalEnviroScreen reports no poverty estimate for 38 of these tracts (the 16 tracts with no residents and 22 others) and no ED visit rates for nine of the tracts without residents [22]. In the file exported from the GIS, these missing values had been recorded as zeros; they were restored as missing for this analysis. The primary sample was therefore the 2,305 tracts with residents and complete data, with no restriction based on park distance. Tracts beyond 1.25 miles by the straight-line measure, approximately the 98th percentile of that measure, were mostly large, sparsely populated peripheral tracts. Restrictions to tracts within 1.25 and within 1.0 mile by that measure were examined as post hoc sensitivity analyses, and these thresholds were chosen after inspection of that measure's distribution.

Statistical analysis

Analyses were performed in R version 4.6.1 with the packages sf (1.1-2), spdep (1.4-2), spatialreg (1.4-3), sandwich (3.1-3), lmtest (0.9-40), MASS (7.3-65), and mgcv (1.9-4), all distributed through the Comprehensive R Archive Network (R Foundation for Statistical Computing, Vienna, Austria). Python 3.14 was used for the shortest-path computation. The data, code, and a log of the models estimated by the analysis code are publicly available (see Additional Information). The primary sample, population weighting, the street-network exposure, and the spatial and ZIP-code analyses were adopted after the initial analysis, whereas the covariates were prespecified. Analyses labeled post hoc (the distance thresholds, the density threshold and tertiles, and the exclusion of the Antelope Valley) and the additional models reported in the Appendices were exploratory. All other analyses were planned to address the stated objectives.

Descriptive statistics were computed for the primary sample, and tracts within and beyond 1.25 miles by the straight-line measure were compared with standardized mean differences and Wilcoxon rank-sum tests. The primary models were population-weighted least-squares regressions of each outcome on park distance, PM2.5, ozone, and poverty (outcome ~ distance + PM2.5 + ozone + poverty). The models were weighted by tract population so that estimates describe the average resident rather than the average tract. Because the outcomes were generated at the ZIP-code level, the 95% confidence intervals (CIs) and p-values for tract-level regressions were based on standard errors clustered by the ZIP code that CalEnviroScreen assigns to each tract, unless stated otherwise. These standard errors were computed with sandwich::vcovCL, using the heteroskedasticity-consistent HC1 small-sample correction and a t distribution with the number of ZIP codes minus one degrees of freedom. For the primary models, p-values from unclustered HC1 standard errors are also reported. Park-distance coefficients are expressed per interquartile range (IQR) of distance (0.376 miles, or 7.5 minutes' walk), and partial R² and standardized coefficients describe the variance associated with park distance.

Residual spatial autocorrelation was assessed with Moran's I test for regression residuals using row-standardized queen contiguity weights. It was present. Additionally, because of how the outcome rates were constructed, dependence was expected in the errors rather than spillover between tracts. Thus, both primary models were re-estimated as population-weighted spatial error models (maximum likelihood, with model-based CIs). A Hausman test was then used to compare the spatial and non-spatial estimates. If the error model is correctly specified, the two estimators agree in expectation. Thus, rejection indicates that the spatial error is correlated with the predictors, which is referred to as spatial confounding.

To locate where any association arose, each predictor was split into its population-weighted ZIP-code mean and the tract's deviation from that mean. Models with ZIP-code fixed effects were also fitted. The share of each variable's (unweighted) variance lying within ZIP codes was computed. Variance inflation factors were below 1.4 for every predictor. Model diagnostics (a residual-versus-fitted plot for the adjusted asthma model and a pairwise correlation matrix) are reported in the Appendices.

Sensitivity analyses varied the sample, the weighting, the spatial weights, the functional form of distance, and the ozone period. The sample was varied in three ways. First, with the straight-line measure as the exposure, models were fitted for all 2,343 tracts and for tracts within 1.25 and within 1.0 mile, each with missing values coded as zero as in the exported file and without population weights. Second, with the primary exposure, models were fitted for tracts within 1.25 and within 1.0 mile by the straight-line measure, which are the post hoc thresholds. Third, models were fitted for tracts with at least 1,000 residents per square mile and for density tertiles, both also defined post hoc. The other analyses used unweighted models, six-nearest-neighbor spatial weights, log2 distance, and CalEnviroScreen 3.0 ozone for 2012-2014 [24], alone and averaged with the 2017-2019 values.

The alternative exposures were the straight-line measure from the tract centroid, as recorded and as recomputed from geometry; straight-line distance from the population-weighted center; street-network and straight-line distance to the single nearest park; street-network distance to open-access parks only and, separately, with overlapping park polygons merged; park acres within 0.5 and 1 mile of the tract centroid; straight-line distance from the tract centroid, and street-network distance from the population-weighted center, to the three nearest parks in the 2016 county park inventory, excluding features with no public access; and the share of the tract within a 2016 half-mile pedestrian-network walkshed of a park. The 2016 inventory and the walksheds came from the 2016 Parks and Recreation Needs Assessment (Los Angeles County Department of Parks and Recreation, Los Angeles, CA, USA). With the 2016 inventory, the parks (2016), the road network (2016), and the outcomes (2015-2017) all come from one period. Walking time at three miles per hour is the network distance rescaled (20 minutes per mile) and gives identical estimates per IQR. A post hoc diagnostic also excluded the Antelope Valley (ZIP codes beginning 935).

Finally, because CalEnviroScreen releases rates rather than counts, count models were possible only at the level at which ED visits are recorded. A negative binomial model of 2015-2017 asthma ED visit counts by ZIP code (California Department of Public Health, Sacramento, CA, USA) was fitted for the ZIP code tabulation areas with published counts for all three years and at least 90% of their population in primary-sample tracts. The model used the logarithm of person-years (three times the 2010 population) as an offset and population-weighted averages of the tract-level exposure and covariates, and it was adjusted for the percentages of residents younger than 10 years and of residents 65 years or older. Residual spatial autocorrelation was assessed, and the model was refitted with a Markov random field spatial effect. A population-weighted least-squares model of the published age-adjusted ZIP-code rates, with HC1 standard errors, was also fitted. Counts below 12 are not published, and no comparable public ZIP-code counts were identified for AMI.

Additional models, reported in the Appendices, were unweighted spatial lag and spatial Durbin models with direct and indirect estimates (CIs and p-values from 1,000 simulations), models with interactions of park distance with poverty and with PM2.5, models additionally adjusted for population density and for the percentage of residents without health insurance (American Community Survey 2015-2019), and models with three-digit ZIP-code fixed effects. Statistical significance was defined as p < 0.05. Generative artificial intelligence (Claude, Anthropic, San Francisco, CA, USA) was used to assist in writing the R and Python code for the statistical analysis and with language and formatting editing.

Ethics

This study analyzed publicly available, de-identified aggregate data at the census tract and ZIP code level and did not involve human or animal subjects; institutional review board review was not required.

## Results

Descriptive characteristics

Across the 2,305 tracts in the primary sample, street-network park distance averaged 0.72 miles with a standard deviation (SD) of 0.46 (median 0.64, IQR 0.47-0.85; range 0.08-8.20), which corresponds to a median walk of 12.8 minutes. The straight-line measure from the tract centroid averaged 0.50 miles (SD 0.33; median 0.44, IQR 0.31-0.59; range 0.03-6.58), and the two measures correlated at 0.83. Asthma ED visit rates averaged 54.2 per 10,000 residents (SD 28.2), and AMI ED visit rates averaged 14.0 per 10,000 (SD 4.8). Mean PM2.5 concentration was 11.7 µg/m³ (SD 1.0), and mean ozone concentration was 0.052 ppm (SD 0.008). On average, 35.1% of tract residents (SD 18.5) lived below twice the federal poverty level (Table 1).

**Table 1:** Descriptive statistics of study variables, primary sample Primary sample: the 2,305 Los Angeles County census tracts with residents and complete data. Street-network distance (the primary exposure) is measured along the road network from the Census 2010 population-weighted tract center; the straight-line measure is measured from the tract's geographic centroid. Both are means over the three nearest recreational spaces. SD: standard deviation; IQR: interquartile range; ED: emergency department; AMI: acute myocardial infarction; PM2.5: fine particulate matter; ppm: parts per million.

Variable	Mean (SD)	Median (IQR)	Range
Street-network distance to the three nearest parks (miles)	0.72 (0.46)	0.64 (0.47-0.85)	0.08-8.20
Walking time to the three nearest parks (minutes, at three miles per hour)	14.5 (9.1)	12.8 (9.5-17.0)	1.6-164.0
Straight-line distance to three nearest parks, from tract centroid (miles)	0.50 (0.33)	0.44 (0.31-0.59)	0.03-6.58
Asthma ED visit rate (per 10,000 residents)	54.2 (28.2)	50.2 (32.8-69.6)	5.9-158.0
AMI ED visit rate (per 10,000 residents)	14.0 (4.8)	13.4 (10.4-17.4)	3.9-27.9
PM2.5 (µg/m³)	11.7 (1.0)	11.9 (11.6-12.1)	5.8-14.6
Ozone (ppm)	0.052 (0.008)	0.050 (0.045-0.059)	0.038-0.069
Residents below twice the federal poverty level (%)	35.1 (18.5)	33.1 (19.1-49.6)	2.7-93.2
Tract population	4,357 (1,507)	4,246 (3,251-5,329)	45-12,463
Population density (residents per square mile)	13,580 (11,276)	10,707 (6,494-17,806)	4-94,111

The 43 tracts in the primary sample with mean straight-line distance beyond 1.25 miles differed markedly from the 2,262 tracts within that distance (Table 2). These tracts had far lower population density (median 566 vs. 10,851 residents per square mile). They also had lower PM2.5, with a standardized mean difference (SMD, within minus beyond) of 2.14, and higher ozone (SMD -1.57). Their median street-network park distance was 1.87 miles, compared with 0.63. Their asthma ED visit rates were higher on average (68.1 vs. 54.0 per 10,000; SMD -0.39; p = 0.097), whereas AMI rates (SMD -0.14) and poverty (SMD 0.16) differed little.

**Table 2:** Tracts within versus beyond 1.25 miles of the three nearest parks by the original straight-line measure, primary sample The 1.25-mile threshold, examined as a post hoc sensitivity analysis, is applied to the straight-line measure (mean straight-line distance from the tract centroid to the three nearest parks). Standardized difference: difference in means divided by the square root of the average of the two variances (within minus beyond). p-values are from Wilcoxon rank-sum tests. Among all 2,343 tracts, 52 lie beyond 1.25 miles; nine of these have no residents or no reported data and are not in the primary sample. n: number of tracts; SD: standard deviation; IQR: interquartile range; ED: emergency department; AMI: acute myocardial infarction; PM2.5: fine particulate matter; ppm: parts per million.

Variable	Within 1.25 miles (n = 2,262), mean (SD)	Within, median (IQR)	Beyond 1.25 miles (n = 43), mean (SD)	Beyond, median (IQR)	Standardized difference	p-value
Street-network distance to the three nearest parks (miles)	0.69 (0.33)	0.63 (0.47-0.83)	2.42 (1.56)	1.87 (1.52-2.72)	-1.53	<0.001
Straight-line distance to the three nearest parks, from tract centroid (miles)	0.47 (0.22)	0.44 (0.31-0.58)	1.99 (1.04)	1.69 (1.37-1.95)	-2.03	<0.001
Asthma ED visit rate (per 10,000 residents)	54.0 (27.8)	50.1 (32.7-69.3)	68.1 (42.9)	57.8 (37.3-83.5)	-0.39	0.097
AMI ED visit rate (per 10,000 residents)	14.0 (4.8)	13.4 (10.4-17.4)	14.7 (4.9)	13.9 (10.7-17.4)	-0.14	0.388
PM2.5 (µg/m³)	11.7 (0.8)	11.9 (11.6-12.1)	8.4 (2.1)	7.5 (6.9-9.5)	2.14	<0.001
Ozone (ppm)	0.051 (0.008)	0.049 (0.045-0.058)	0.062 (0.005)	0.062 (0.062-0.064)	-1.57	<0.001
Residents below twice the federal poverty level (%)	35.2 (18.5)	33.1 (19.1-49.7)	32.6 (14.3)	33.1 (20.6-40.9)	0.16	0.514
Tract population	4,356 (1,476)	4,250 (3,255-5,316)	4,419 (2,691)	4,080 (2,392-6,017)	-0.03	1
Population density (residents per square mile)	13,793 (11,263)	10,851 (6,671-17,901)	2,336 (3,703)	566 (118-2,701)	1.37	<0.001

Spatial patterns

Asthma and AMI ED visit rates showed similar spatial patterns: the highest rates of both concentrated in the urban core, with additional elevated areas in the San Fernando Valley and the Antelope Valley, while coastal and mountainous tracts generally showed lower rates (Figures 2A, 2B). The two pollutants showed nearly opposite spatial patterns (Figures 3A, 3B). Ozone increased from the coast toward inland and valley areas, consistent with its regional photochemical origin. Meanwhile, PM2.5 concentrated in the dense southern urban areas where local emission sources are greatest. High-poverty tracts appeared as scattered pockets across the county but clustered in the urban core and parts of the inland county, overlapping substantially with the areas of highest ED visit rates (Figure 4). Park distance was spatially heterogeneous by either measure: extreme distances occurred almost exclusively in large peripheral tracts, and tracts with relatively good and relatively poor park access were interspersed throughout the rest of the county (Figures 5A, 5B). Of all 2,343 mapped tracts, the 52 beyond 1.25 miles by the straight-line measure lay overwhelmingly along the county's periphery (Figure 5C). All study variables were strongly spatially clustered in the primary sample (Moran's I 0.82 for asthma, 0.76 for AMI, 0.49 for park distance, 0.98 for PM2.5, 0.995 for ozone, and 0.70 for poverty).

**Figure 2:**
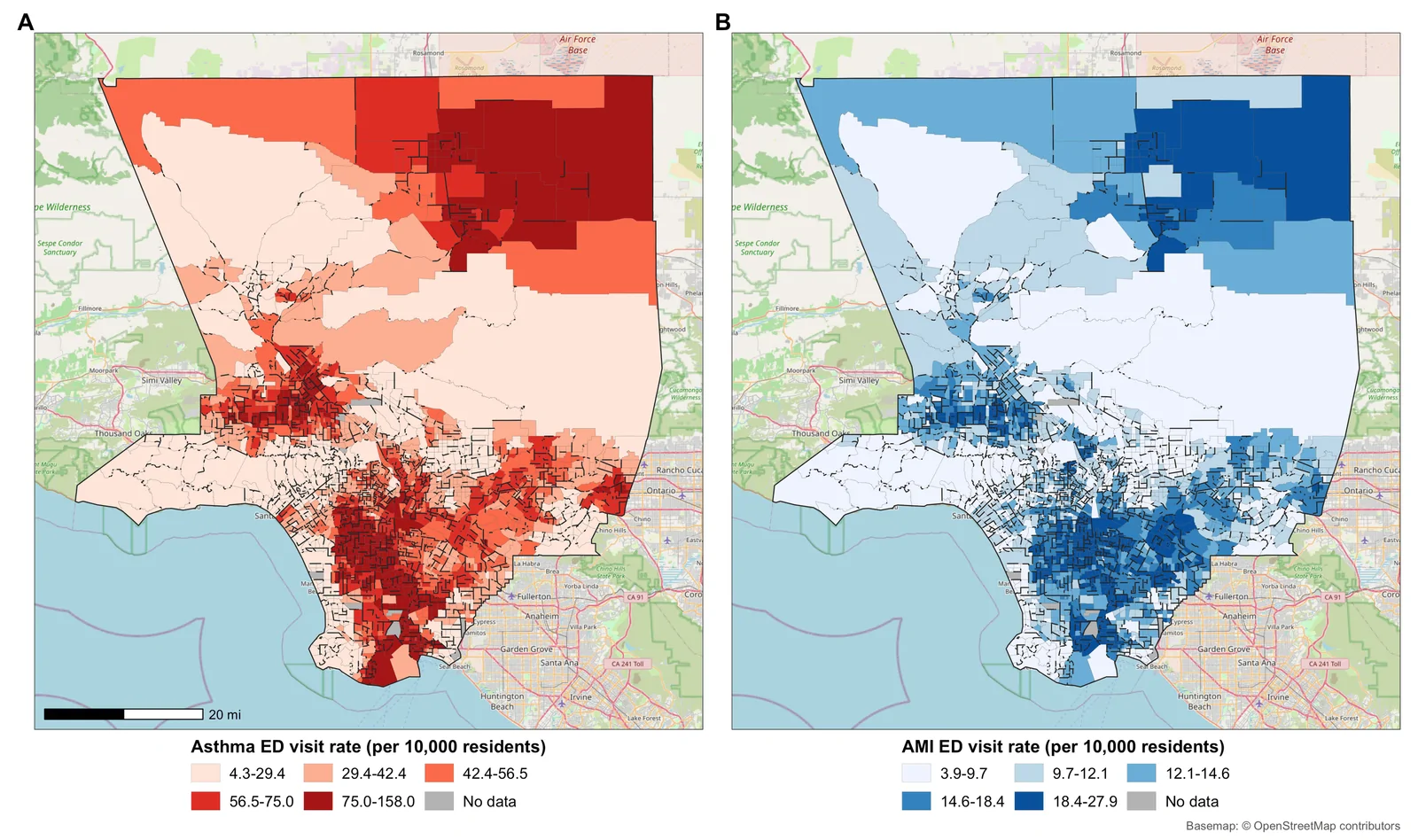
Age-adjusted asthma and acute myocardial infarction emergency department visit rates by census tract (A) Asthma ED visit rates per 10,000 residents. (B) AMI ED visit rates per 10,000 residents. Rates are age-adjusted 2015-2017 averages, classified by quantiles. All 2,343 county tracts are mapped; the nine tracts for which CalEnviroScreen reports no rate are shown as "No data." Data are from CalEnviroScreen 4.0. AMI: acute myocardial infarction; ED: emergency department. Basemap: © OpenStreetMap contributors, available under the Open Database License (https://www.openstreetmap.org/copyright) [23]; reproduced under that license, which requires attribution only.

**Figure 3:**
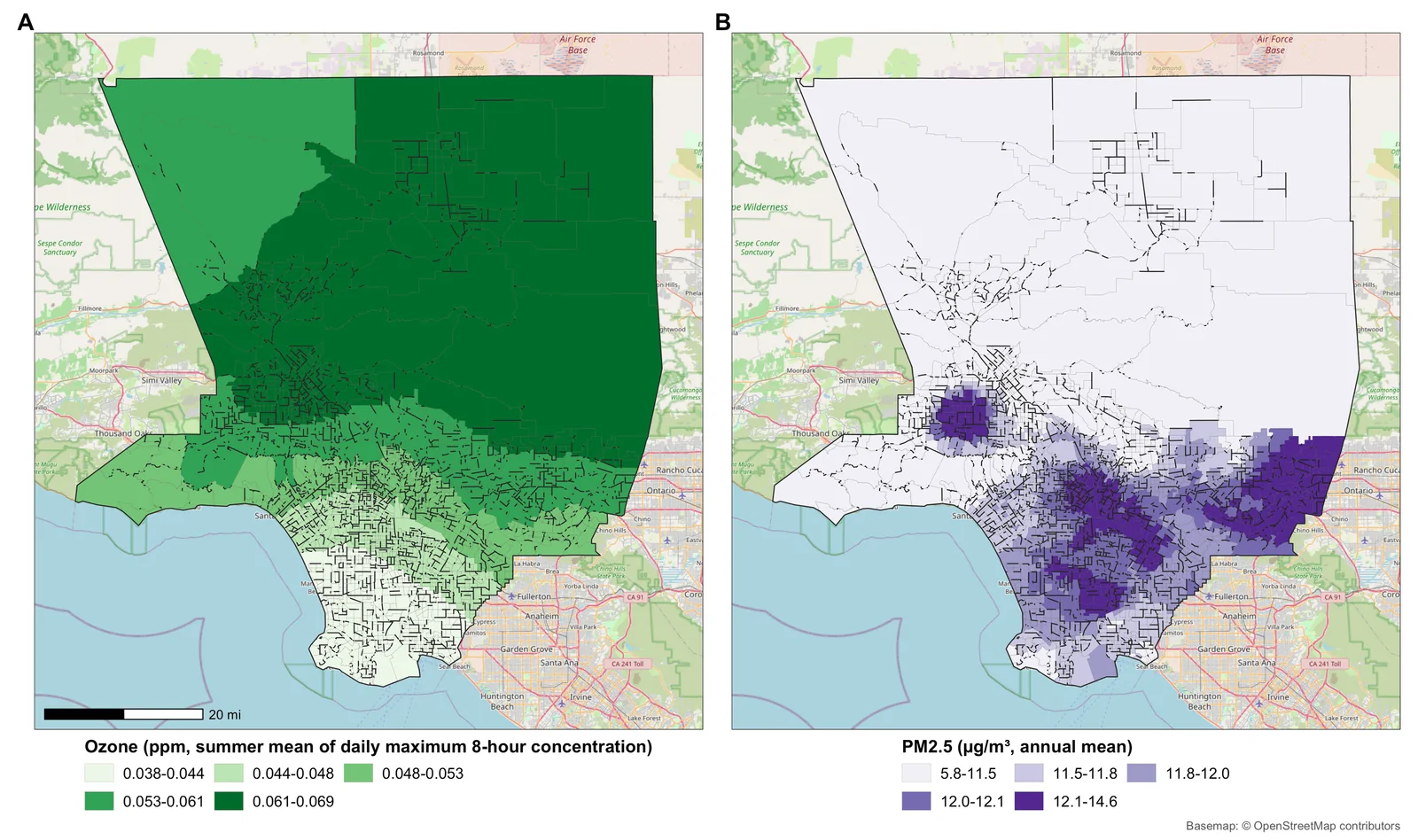
Ozone and PM2.5 concentrations by census tract (A) Summer-season mean of daily maximum 8-hour ozone concentration (ppm, 2017-2019). (B) Annual mean PM2.5 concentration (µg/m³, 2015-2017). Quantile classification. All 2,343 county tracts are mapped. Data are from CalEnviroScreen 4.0. ppm: parts per million; PM2.5: fine particulate matter no larger than 2.5 micrometers in diameter. Basemap: © OpenStreetMap contributors, available under the Open Database License (https://www.openstreetmap.org/copyright) [23]; reproduced under that license, which requires attribution only.

**Figure 4:**
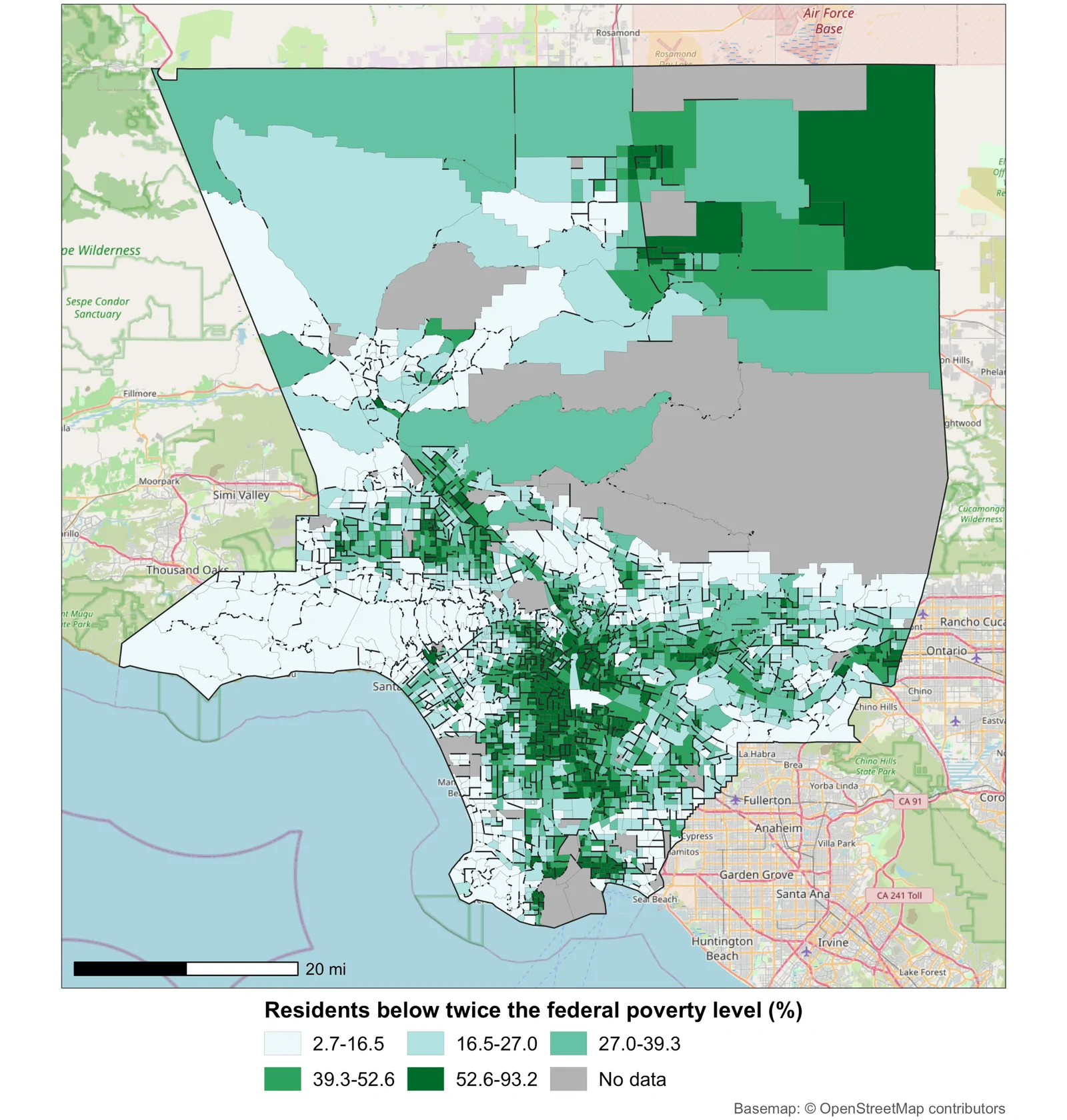
Residents living below twice the federal poverty level by census tract Percentage of residents living below twice the federal poverty level (American Community Survey 2015-2019 estimates), classified by quantiles. All 2,343 county tracts are mapped; the 38 tracts for which CalEnviroScreen reports no estimate are shown as "No data." Data are from CalEnviroScreen 4.0. Basemap: © OpenStreetMap contributors, available under the Open Database License (https://www.openstreetmap.org/copyright) [23]; reproduced under that license, which requires attribution only.

**Figure 5:**
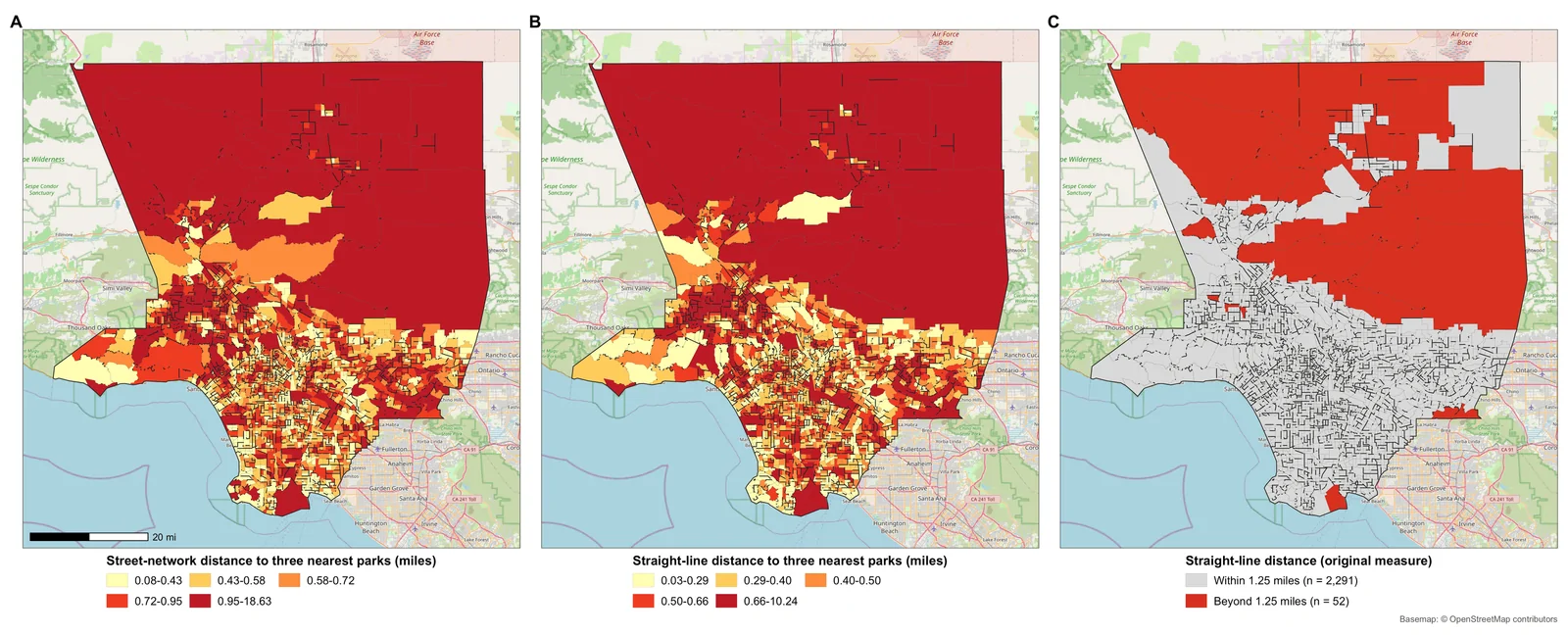
Park distance by census tract (A) The primary exposure: mean distance along the street network from each tract's Census 2010 population-weighted center to the nearest boundary of each of the three nearest recreational spaces in the county inventory. (B) The straight-line measure: mean straight-line distance from each tract centroid to the three recreational spaces nearest by straight-line distance. (C) The 52 tracts beyond 1.25 miles by the straight-line measure, shown in red; this is the post hoc distance restriction examined in sensitivity analyses (43 of the 52 are in the primary sample). All 2,343 tracts are mapped; quantile classification. n: number of tracts. Basemap: © OpenStreetMap contributors, available under the Open Database License (https://www.openstreetmap.org/copyright) [23]; reproduced under that license, which requires attribution only.

Bivariate associations

In population-weighted bivariate models, street-network park distance was essentially unrelated to either outcome (asthma: 2.33 per 10,000 per mile, or 0.87 per IQR, 95% CI -7.35 to 12.01 per mile, p = 0.636, R² = 0.001; AMI: -0.17 per mile, or -0.06 per IQR, 95% CI -1.35 to 1.01 per mile, p = 0.775) (Table 3). Tracts farther from parks had lower PM2.5 (-1.07 µg/m³ per mile, 95% CI -1.48 to -0.65, p < 0.001; R² = 0.186), higher ozone (0.008 ppm per mile, 95% CI 0.006 to 0.010, p < 0.001; R² = 0.155), and lower poverty (-5.55 percentage points per mile, 95% CI -9.18 to -1.92, p = 0.003; R² = 0.016). In bivariate models of the outcomes on each pollutant, PM2.5 was not associated with either outcome (asthma -3.12 per µg/m³, p = 0.385; AMI 0.08, p = 0.859). In contrast, ozone was negatively associated with AMI (-0.97 per 0.01 ppm, p = 0.008), but it was not associated with asthma (-3.28 per 0.01 ppm, p = 0.211). With the straight-line measure from the tract centroid, park distance was not statistically significantly associated with poverty in the same weighted model (-1.37 percentage points per mile, 95% CI -5.38 to 2.64, p = 0.502). In unweighted models with conventional standard errors, this association was negative across all 2,343 tracts when missing poverty values were coded as zero (-3.30 percentage points per mile, 95% CI -5.19 to -1.41, p < 0.001). However, when those values were treated as missing, the estimate was -1.24 (95% CI -3.54 to 1.05, p = 0.287). Model diagnostics and scatterplots are provided in Appendix 1.

**Table 3:** Bivariate associations between street-network park distance and each study variable Each row is a separate population-weighted least-squares regression of the variable on park distance (mean street-network distance from the population-weighted tract center to the three nearest parks) in the primary sample (n = 2,305). The IQR of park distance is 0.376 miles. CIs and p-values use standard errors clustered by ZIP code. CI: confidence interval; IQR: interquartile range; ED: emergency department; AMI: acute myocardial infarction; PM2.5: fine particulate matter; ppm: parts per million.

Variable	Coefficient per mile of park distance	95% CI (per mile)	Coefficient per IQR of park distance	p-value	R²
Asthma ED visits (per 10,000)	2.33	-7.35 to 12.01	0.87	0.636	0.001
AMI ED visits (per 10,000)	-0.17	-1.35 to 1.01	-0.06	0.775	0
PM2.5 (µg/m³)	-1.07	-1.48 to -0.65	-0.40	<0.001	0.186
Ozone (ppm)	0.008	0.0063 to 0.0096	0.003	<0.001	0.155
Poverty (percentage points)	-5.55	-9.18 to -1.92	-2.08	0.003	0.016

Adjusted models

After adjustment for PM2.5, ozone, and poverty, park distance had positive coefficients for both outcomes in the population-weighted models (Table 4). An IQR increase in park distance (0.376 miles) corresponded to 1.64 more asthma ED visits per 10,000 residents (95% CI -0.14 to 3.41, p = 0.070), which was not statistically significant, and to 0.34 more AMI ED visits per 10,000 (95% CI 0.06 to 0.63, p = 0.018). These estimates correspond to 4.36 and 0.92 per mile. With unclustered HC1 standard errors, which do not account for the ZIP-code level at which the outcomes were generated, the corresponding p-values are 0.006 and < 0.001. Park distance accounted for less than 1% of the residual variation in each outcome (partial R² 0.004 and 0.006), whereas poverty showed the strongest association in both models (standardized coefficient 0.56 for asthma and 0.51 for AMI, compared with 0.06 and 0.08 for park distance; partial R² 0.31 and 0.26). Both pollutants had negative coefficients in these models (asthma: PM2.5 -5.49 per µg/m³, 95% CI -10.70 to -0.29; ozone -3.81 per 0.01 ppm, 95% CI -7.46 to -0.17).

**Table 4:** Adjusted models of asthma and acute myocardial infarction emergency department visit rates Both models are population-weighted and use the primary sample (n = 2,305); outcomes are ED visits per 10,000 residents; park distance is the mean street-network distance from the population-weighted tract center to the three nearest parks. Weighted least squares: CIs and p-values from standard errors clustered by ZIP code (279 ZIP codes; park distance with unclustered HC1 standard errors: p = 0.006 for asthma and p < 0.001 for AMI); R² 0.333 (asthma) and 0.287 (AMI); partial R² of park distance 0.004 and 0.006; residual Moran's I 0.652 and 0.596 (both p < 0.001). Spatial error model: maximum likelihood with queen contiguity weights and model-based CIs; spatial parameter λ 0.899 and 0.835; Hausman test of spatial versus non-spatial estimates p < 0.001 for both. Per mile, the park-distance coefficients are 4.36 (asthma) and 0.92 (AMI) in the weighted least-squares models. IQR: interquartile range; CI: confidence interval; ED: emergency department; AMI: acute myocardial infarction; PM2.5: fine particulate matter; ppm: parts per million; HC1: heteroskedasticity-consistent standard errors with small-sample correction.

Model	Predictor	Asthma, coefficient	Asthma, 95% CI	Asthma, p-value	AMI, coefficient	AMI, 95% CI	AMI, p-value
Weighted least squares	Park distance (per IQR, 0.376 miles)	1.64	-0.14 to 3.41	0.070	0.34	0.06 to 0.63	0.018
Weighted least squares	PM2.5 (per µg/m³)	-5.49	-10.70 to -0.29	0.039	-0.33	-0.94 to 0.27	0.279
Weighted least squares	Ozone (per 0.01 ppm)	-3.81	-7.46 to -0.17	0.040	-0.87	-1.42 to -0.32	0.002
Weighted least squares	Poverty (per percentage point)	0.88	0.73 to 1.02	<0.001	0.14	0.11 to 0.16	<0.001
Weighted least squares	Intercept	104.9	36.53 to 173.26	0.003	17.17	8.51 to 25.82	<0.001
Spatial error model	Park distance (per IQR, 0.376 miles)	-1.01	-1.61 to -0.41	<0.001	-0.12	-0.25 to 0.00	0.054
Spatial error model	PM2.5 (per µg/m³)	0.53	-2.72 to 3.77	0.751	0.18	-0.34 to 0.70	0.496
Spatial error model	Ozone (per 0.01 ppm)	-1.87	-7.05 to 3.32	0.481	-0.69	-1.41 to 0.04	0.063
Spatial error model	Poverty (per percentage point)	0.15	0.10 to 0.19	<0.001	0.03	0.02 to 0.04	<0.001
Spatial error model	Intercept	57.02	6.52 to 107.51	0.027	15.08	7.12 to 23.04	<0.001

Residuals of both models were strongly spatially autocorrelated (Moran's I 0.652 for asthma and 0.596 for AMI; both p < 0.001). In population-weighted spatial error models, which account for this dependence, the park-distance coefficient became negative for both outcomes (asthma: -1.01 per IQR, 95% CI -1.61 to -0.41, p < 0.001; AMI: -0.12 per IQR, 95% CI -0.25 to 0.00, p = 0.054). The PM2.5 coefficient became positive and not statistically significant, and poverty remained positively associated with both outcomes (asthma 0.15 per percentage point, 95% CI 0.10 to 0.19; AMI 0.03, 95% CI 0.02 to 0.04; both p < 0.001) (Table 4). A Hausman test indicated that the spatial and non-spatial estimates differed systematically (both p < 0.001). This points to spatial confounding, so neither estimate can be read as unconfounded.

Within and between ZIP codes

Only 9.5% of the tract-level variance in asthma ED visit rates and 17.1% of that in AMI rates lay within ZIP codes, compared with 53.9% of the variance in park distance (279 ZIP codes; median seven tracts per ZIP code). The positive coefficients arose from differences between ZIP codes. The between-ZIP coefficient was 4.64 asthma visits per 10,000 per IQR (95% CI 0.22 to 9.05, p = 0.040) and 1.05 AMI visits (95% CI 0.34 to 1.77, p = 0.004). In contrast, the within-ZIP coefficients were close to zero (asthma -0.29, 95% CI -0.82 to 0.24; AMI -0.09, 95% CI -0.20 to 0.03), as were the coefficients in models with ZIP-code fixed effects (Table 5). PM2.5 showed the opposite pattern: its coefficient was positive within ZIP codes (asthma 4.03 per µg/m³, 95% CI 1.40 to 6.67, p = 0.003; AMI 0.89, 95% CI 0.23 to 1.55, p = 0.009) and negative between them for asthma (-5.16, 95% CI -9.85 to -0.47, p = 0.031).

**Table 5:** Park distance and emergency department visit rates within and between ZIP codes Population-weighted least-squares models in the primary sample (n = 2,305; 279 ZIP codes), adjusted for PM2.5, ozone, and poverty, each also split into ZIP-code mean and within-ZIP deviation; CIs and p-values from standard errors clustered by ZIP code. Share of tract-level (unweighted) variance lying within ZIP codes: asthma 9.5%, AMI 17.1%, park distance 53.9%, PM2.5 2.8%, ozone 0.7%, poverty 34.1%. IQR: interquartile range (0.376 miles); CI: confidence interval; AMI: acute myocardial infarction; PM2.5: fine particulate matter; ZIP: Zone Improvement Plan.

Outcome	Component of park distance	Coefficient per IQR	95% CI	p-value
Asthma	Between ZIP codes (ZIP-code mean distance)	4.64	0.22 to 9.05	0.04
Asthma	Within ZIP codes (deviation from ZIP-code mean)	-0.29	-0.82 to 0.24	0.277
Asthma	ZIP-code fixed effects	-0.29	-0.86 to 0.27	0.307
AMI	Between ZIP codes (ZIP-code mean distance)	1.05	0.34 to 1.77	0.004
AMI	Within ZIP codes (deviation from ZIP-code mean)	-0.09	-0.20 to 0.03	0.130
AMI	ZIP-code fixed effects	-0.09	-0.20 to 0.03	0.155

Sensitivity analyses

In non-spatial models, the park-distance coefficient for asthma was positive in every distance-based sensitivity analysis except the lowest density tertile (Table 6). However, apart from the unweighted models with missing values coded as zero (described below), it was statistically significant only in the following analyses. When the sample was redefined, it was statistically significant among tracts with at least 1,000 residents per square mile (3.09 per IQR, 95% CI 0.99 to 5.20), in the middle and highest density tertiles (2.99 and 5.69), and when tracts beyond 1.25 or 1.0 mile by the straight-line measure were excluded (2.34, 95% CI 0.51 to 4.17, and 2.02, 95% CI 0.02 to 4.01). When distance was redefined, it was statistically significant with log2 distance (3.23 per doubling), with street-network distance to the 2016 county park inventory (1.84, 95% CI 0.02 to 3.66), and with the straight-line measures: the measure from the tract centroid (2.40, 95% CI 0.41 to 4.39), the same measure recomputed from geometry (2.51), straight-line distance from the population-weighted center (3.49) or to the single nearest park (2.40), and straight-line distance to the 2016 county park inventory (2.70).

**Table 6:** Sensitivity analyses for asthma emergency department visit rates Unless stated otherwise, models are population-weighted, use the primary sample, adjust for PM2.5, ozone, and poverty, and use the primary exposure (mean street-network distance from the Census 2010 population-weighted tract center to the three nearest parks, measured on the 2016 TIGER/Line road network, excluding limited-access highways and ramps). Coefficients are per interquartile range (IQR) in the primary sample: 0.376 miles of street-network distance in the primary, sample-definition, weighting, spatial-weights, and ozone rows; 0.279 miles of straight-line distance in the row 'Straight-line measure: distance from tract centroid to three nearest parks' and the three rows with missing values coded as zero; and each alternative exposure's own IQR in the other exposure rows (straight-line measure recomputed from geometry 0.285 miles; straight-line distance from the population-weighted center 0.281 miles; street-network distance to the single nearest park 0.385 miles, to open-access parks only 0.415 miles, with overlapping polygons merged 0.380 miles, and to the 2016 inventory 0.381 miles; straight-line distance to the single nearest park 0.282 miles; park acres within 0.5 mile 18.3 acres and within 1 mile 87.8 acres; straight-line distance to the 2016 inventory 0.293 miles; walkshed share 0.64); the log2 row is per doubling of distance. For park acres and walkshed share, negative values mean that more park access is associated with lower rates. Non-spatial CIs and p-values use standard errors clustered by ZIP code; the spatial model is a spatial error model with queen contiguity weights (six nearest neighbors where stated). The density threshold and tertiles were defined post hoc; tertiles were not fitted with spatial models (cells marked -). n: number of tracts; CI: confidence interval; PM2.5: fine particulate matter; TIGER: Topologically Integrated Geographic Encoding and Referencing.

Analysis	n	Coefficient	95% CI	p-value	R²	Standardized coefficient	Spatial model coefficient	Spatial model 95% CI	Spatial model p-value
Primary analysis (population-weighted; street-network distance)	2,305	1.64	-0.14 to 3.41	0.070	0.333	0.062	-1.01	-1.61 to -0.41	<0.001
Straight-line measure: distance from tract centroid to three nearest parks	2,305	2.4	0.41 to 4.39	0.018	0.337	0.094	-1.28	-1.87 to -0.69	<0.001
Straight-line measure, all 2,343 tracts, missing values coded as zero, unweighted	2,343	1.38	0.25 to 2.51	0.017	0.316	0.07	-1.29	-1.76 to -0.82	<0.001
Straight-line measure, tracts within 1.25 miles (n = 2,291), missing values coded as zero, unweighted	2,291	3.87	1.86 to 5.88	<0.001	0.316	0.108	-0.76	-1.64 to 0.12	0.089
Straight-line measure, tracts within 1.0 mile (n = 2,237), missing values coded as zero, unweighted	2,237	3.91	1.67 to 6.15	<0.001	0.317	0.1	-0.64	-1.57 to 0.29	0.180
Primary sample, unweighted	2,305	0.91	-0.73 to 2.56	0.275	0.305	0.039	-0.43	-0.96 to 0.10	0.108
Tracts within 1.25 miles by the straight-line measure (post hoc threshold)	2,262	2.34	0.51 to 4.17	0.012	0.323	0.072	-0.63	-1.37 to 0.11	0.098
Tracts within 1.0 mile by the straight-line measure (post hoc threshold)	2,210	2.02	0.02 to 4.01	0.048	0.319	0.058	-0.41	-1.19 to 0.38	0.313
At least 1,000 residents per square mile (post hoc)	2,224	3.09	0.99 to 5.20	0.004	0.331	0.096	-0.9	-1.70 to -0.10	0.028
Lowest density tertile	769	-0.17	-1.67 to 1.33	0.820	0.486	-0.009	-	-	-
Middle density tertile	768	2.99	0.45 to 5.52	0.021	0.227	0.09	-	-	-
Highest density tertile	768	5.69	0.62 to 10.76	0.028	0.243	0.141	-	-	-
Six-nearest-neighbor spatial weights	2,305	1.64	-0.14 to 3.41	0.070	0.333	0.062	-0.96	-1.57 to -0.34	0.002
Excluding the Antelope Valley (post hoc)	2,227	1.43	-0.18 to 3.03	0.081	0.315	0.048	-0.46	-1.16 to 0.25	0.203
Log2 of street-network distance (per doubling)	2,305	3.23	0.89 to 5.57	0.007	0.336	0.082	-0.7	-1.64 to 0.23	0.142
Straight-line measure recomputed from geometry	2,305	2.51	0.45 to 4.57	0.017	0.338	0.096	-1.2	-1.81 to -0.58	<0.001
Straight-line distance from population-weighted tract center	2,305	3.49	1.18 to 5.79	0.003	0.342	0.118	-1.24	-1.95 to -0.53	<0.001
Street-network distance to the single nearest park	2,305	1.57	-0.05 to 3.20	0.057	0.333	0.052	-0.64	-1.19 to -0.09	0.022
Street-network distance to open-access parks only	2,305	1.57	-0.36 to 3.49	0.110	0.333	0.056	-0.9	-1.53 to -0.26	0.006
Street-network distance with overlapping park polygons merged	2,305	0.85	-0.81 to 2.51	0.315	0.331	0.034	-1	-1.57 to -0.43	<0.001
Straight-line distance from tract centroid to the single nearest park	2,305	2.4	0.63 to 4.18	0.008	0.336	0.079	-0.75	-1.33 to -0.17	0.012
Park acres within 0.5 mile of tract centroid	2,305	-0.71	-1.11 to -0.31	<0.001	0.335	-0.072	-0.11	-0.28 to 0.06	0.193
Park acres within 1 mile of tract centroid	2,305	-1.26	-1.91 to -0.61	<0.001	0.339	-0.096	-0.09	-0.38 to 0.19	0.522
Straight-line distance from tract centroid to 2016 county park inventory	2,305	2.7	0.42 to 4.98	0.021	0.338	0.098	-1.01	-1.65 to -0.38	0.002
Street-network distance to 2016 county park inventory	2,305	1.84	0.02 to 3.66	0.047	0.334	0.069	-0.81	-1.41 to -0.20	0.009
Share of tract within a 2016 half-mile walkshed	2,305	-5.06	-8.35 to -1.77	0.003	0.338	-0.093	0.71	-0.49 to 1.91	0.246
Ozone from CalEnviroScreen 3.0 (2012-2014)	2,305	1.25	-0.47 to 2.96	0.153	0.326	0.047	-1.02	-1.62 to -0.42	<0.001
Ozone averaged over CalEnviroScreen 3.0 and 4.0	2,305	1.51	-0.23 to 3.26	0.089	0.33	0.058	-1.02	-1.62 to -0.42	<0.001

Meanwhile, the asthma coefficient was smaller and not statistically significant in several other analyses. These included the model without population weights (0.91, p = 0.275), the model that excluded the Antelope Valley (1.43, p = 0.081), and the lowest density tertile (-0.17, p = 0.820). They also included street-network distance to the single nearest park (1.57, p = 0.057), to open-access parks only (1.57, p = 0.110), or with overlapping park polygons merged (0.85, p = 0.315), as well as the earlier CalEnviroScreen 3.0 ozone values, alone or averaged with the 2017-2019 values (1.25 and 1.51). The earlier ozone values correlated 0.93 with the 2017-2019 values across tracts. Additionally, the interaction between park distance and density tertile was not statistically significant (asthma p = 0.231; AMI p = 0.420).

For AMI, the coefficient was positive and statistically significant in most analyses (Table 7), including the straight-line measure from the tract centroid (0.40 per IQR, 95% CI 0.10 to 0.71), open-access parks only (0.41, 95% CI 0.10 to 0.71), and street-network distance to the 2016 county park inventory (0.37, 95% CI 0.07 to 0.67). It was not statistically significant without weights (0.22, p = 0.067), in the lowest and highest density tertiles, with street-network distance to the single nearest park (0.26, p = 0.053), or with overlapping polygons merged (0.23, p = 0.079).

**Table 7:** Sensitivity analyses for acute myocardial infarction emergency department visit rates Unless stated otherwise, models are population-weighted, use the primary sample, adjust for PM2.5, ozone, and poverty, and use the primary exposure (mean street-network distance from the Census 2010 population-weighted tract center to the three nearest parks, measured on the 2016 TIGER/Line road network, excluding limited-access highways and ramps). Coefficients are per interquartile range (IQR) in the primary sample: 0.376 miles of street-network distance in the primary, sample-definition, weighting, spatial-weights, and ozone rows; 0.279 miles of straight-line distance in the row 'Straight-line measure: distance from tract centroid to three nearest parks' and the three rows with missing values coded as zero; and each alternative exposure's own IQR in the other exposure rows (straight-line measure recomputed from geometry 0.285 miles; straight-line distance from the population-weighted center 0.281 miles; street-network distance to the single nearest park 0.385 miles, to open-access parks only 0.415 miles, with overlapping polygons merged 0.380 miles, and to the 2016 inventory 0.381 miles; straight-line distance to the single nearest park 0.282 miles; park acres within 0.5 mile 18.3 acres and within 1 mile 87.8 acres; straight-line distance to the 2016 inventory 0.293 miles; walkshed share 0.64); the log2 row is per doubling of distance. For park acres and walkshed share, negative values mean that more park access is associated with lower rates. Non-spatial CIs and p-values use standard errors clustered by ZIP code; the spatial model is a spatial error model with queen contiguity weights (six nearest neighbors where stated). The density threshold and tertiles were defined post hoc; tertiles were not fitted with spatial models (cells marked -). n: number of tracts; CI: confidence interval; PM2.5: fine particulate matter; TIGER: Topologically Integrated Geographic Encoding and Referencing.

Analysis	n	Coefficient	95% CI	p-value	R²	Standardized coefficient	Spatial model coefficient	Spatial model 95% CI	Spatial model p-value
Primary analysis (population-weighted; street-network distance)	2,305	0.34	0.06 to 0.63	0.018	0.287	0.078	-0.12	-0.25 to 0.00	0.054
Straight-line measure: distance from tract centroid to three nearest parks	2,305	0.4	0.10 to 0.71	0.01	0.29	0.094	-0.15	-0.27 to -0.02	0.021
Straight-line measure, all 2,343 tracts, missing values coded as zero, unweighted	2,343	0.22	0.03 to 0.40	0.021	0.269	0.064	-0.18	-0.28 to -0.07	<0.001
Straight-line measure, tracts within 1.25 miles (n = 2,291), missing values coded as zero, unweighted	2,291	0.6	0.25 to 0.95	<0.001	0.272	0.096	-0.17	-0.36 to 0.02	0.085
Straight-line measure, tracts within 1.0 mile (n = 2,237), missing values coded as zero, unweighted	2,237	0.58	0.19 to 0.97	0.004	0.277	0.085	-0.1	-0.31 to 0.10	0.305
Primary sample, unweighted	2,305	0.22	-0.02 to 0.45	0.067	0.259	0.056	-0.06	-0.17 to 0.05	0.274
Tracts within 1.25 miles by the straight-line measure (post hoc threshold)	2,262	0.48	0.15 to 0.81	0.005	0.285	0.086	-0.06	-0.22 to 0.10	0.445
Tracts within 1.0 mile by the straight-line measure (post hoc threshold)	2,210	0.42	0.06 to 0.78	0.021	0.287	0.071	-0.02	-0.19 to 0.14	0.785
At least 1,000 residents per square mile (post hoc)	2,224	0.6	0.26 to 0.94	<0.001	0.286	0.111	-0.11	-0.28 to 0.06	0.196
Lowest density tertile	769	0.03	-0.19 to 0.25	0.782	0.362	0.011	-	-	-
Middle density tertile	768	0.74	0.25 to 1.22	0.003	0.202	0.133	-	-	-
Highest density tertile	768	0.81	-0.12 to 1.73	0.087	0.203	0.106	-	-	-
Six-nearest-neighbor spatial weights	2,305	0.34	0.06 to 0.63	0.018	0.287	0.078	-0.14	-0.27 to -0.01	0.032
Excluding the Antelope Valley (post hoc)	2,227	0.36	0.05 to 0.66	0.023	0.293	0.066	-0.09	-0.25 to 0.06	0.228
Log2 of street-network distance (per doubling)	2,305	0.67	0.24 to 1.10	0.002	0.291	0.101	-0.08	-0.27 to 0.12	0.426
Straight-line measure recomputed from geometry	2,305	0.43	0.11 to 0.75	0.008	0.29	0.098	-0.13	-0.26 to 0.00	0.051
Straight-line distance from population-weighted tract center	2,305	0.55	0.20 to 0.91	0.002	0.293	0.111	-0.09	-0.24 to 0.06	0.223
Street-network distance to the single nearest park	2,305	0.26	-0.003 to 0.52	0.053	0.285	0.051	-0.09	-0.20 to 0.03	0.141
Street-network distance to open-access parks only	2,305	0.41	0.10 to 0.71	0.009	0.288	0.086	-0.1	-0.24 to 0.03	0.131
Street-network distance with overlapping park polygons merged	2,305	0.23	-0.03 to 0.49	0.079	0.285	0.056	-0.13	-0.25 to -0.01	0.030
Straight-line distance from tract centroid to the single nearest park	2,305	0.4	0.12 to 0.68	0.005	0.288	0.079	-0.07	-0.19 to 0.05	0.274
Park acres within 0.5 mile of tract centroid	2,305	-0.12	-0.18 to -0.05	<0.001	0.288	-0.072	-0.05	-0.08 to -0.01	0.008
Park acres within 1 mile of tract centroid	2,305	-0.2	-0.31 to -0.09	<0.001	0.29	-0.092	-0.08	-0.13 to -0.02	0.013
Straight-line distance from tract centroid to 2016 county park inventory	2,305	0.49	0.13 to 0.85	0.008	0.292	0.106	-0.09	-0.22 to 0.04	0.180
Street-network distance to 2016 county park inventory	2,305	0.37	0.07 to 0.67	0.016	0.288	0.084	-0.08	-0.21 to 0.05	0.206
Share of tract within a 2016 half-mile walkshed	2,305	-0.68	-1.24 to -0.11	0.019	0.287	-0.074	0.24	-0.01 to 0.49	0.061
Ozone from CalEnviroScreen 3.0 (2012-2014)	2,305	0.27	0.01 to 0.54	0.039	0.276	0.062	-0.13	-0.26 to -0.005	0.042
Ozone averaged over CalEnviroScreen 3.0 and 4.0	2,305	0.32	0.05 to 0.60	0.021	0.282	0.073	-0.13	-0.25 to -0.0005	0.049

In the conventional models, more park acreage within 0.5 or 1 mile of the tract centroid (for asthma, -0.71 and -1.26 per IQR of 18.3 and 87.8 acres) and a larger share of the tract within a 2016 half-mile walkshed (-5.06 per IQR of 0.64) were associated with lower rates of both outcomes. In the unweighted models with missing values coded as zero, the straight-line measure gave 3.87 per IQR for asthma within 1.25 miles (13.85 per mile) and 3.91 within 1.0 mile (13.99 per mile), compared with 1.38 in all 2,343 tracts (95% CI 0.25 to 2.51).

In the spatial error models (Tables 6, 7), no distance measure had a statistically significant positive coefficient for either outcome in any sample or with either set of spatial weights. For asthma, the coefficients were negative and statistically significant for the primary exposure (-1.01, 95% CI -1.61 to -0.41), for the straight-line measure from the tract centroid (-1.28), and for every other distance measure except log2 distance. This included street-network distance to the 2016 county park inventory (-0.81, 95% CI -1.41 to -0.20). For AMI, the coefficients were negative for every distance measure. Among the alternative distance measures, the AMI coefficients were statistically significant only for the straight-line measure from the tract centroid (-0.15) and the merged-polygon measure (-0.13). For the primary exposure, the AMI coefficient was -0.12 (95% CI -0.25 to 0.00, p = 0.054). This coefficient was statistically significant with six-nearest-neighbor weights (-0.14, p = 0.032) and with the earlier or averaged ozone values (-0.13 with each; p = 0.042 and p = 0.049). The AMI coefficient for the straight-line measure was also statistically significant in the unweighted model of all 2,343 tracts with missing values coded as zero (-0.18, p < 0.001).

The park-area measures pointed in different directions in the spatial error models (Tables 6, 7). More park acreage within 0.5 or 1 mile was associated with lower AMI rates (-0.05 and -0.08 per IQR; p = 0.008 and p = 0.013), whereas the walkshed share had positive coefficients that were not statistically significant for either outcome (0.71 and 0.24). The negative spatial coefficient for asthma in the primary sample was largely attributable to the Antelope Valley. After its exclusion, the asthma coefficient was -0.46 (95% CI -1.16 to 0.25), and the AMI coefficient, which changed less, was -0.09 (95% CI -0.25 to 0.06). In the unweighted model with missing values coded as zero and the straight-line measure within 1.25 miles, the spatial error coefficient for asthma was -0.76 (95% CI -1.64 to 0.12).

ZIP-code counts

Across 259 ZIP code tabulation areas with 150,512 asthma ED visits in 2015-2017, a negative binomial model of unsmoothed counts showed a rate ratio of 1.09 per IQR of ZIP-level park distance (0.292 miles; 95% CI 1.03 to 1.16, p = 0.006). However, its residuals were spatially autocorrelated (Moran's I 0.33, p < 0.001), and with a spatial random effect, the rate ratio was 0.99 (95% CI 0.93 to 1.04, p = 0.631). A population-weighted least-squares model of the published age-adjusted ZIP-code rates gave 4.18 more visits per 10,000 per IQR (95% CI 0.70 to 7.65, p = 0.019) (Table 8). Poverty was positively associated with asthma visits in both count models (rate ratio 1.027 per percentage point of residents below twice the federal poverty level without the spatial effect and 1.025 with it; both p < 0.001).

**Table 8:** Park distance and asthma emergency department visits at the ZIP-code level, 2015-2017 Two hundred and fifty-nine ZIP code tabulation areas with published counts for all three years and at least 90% of their population in primary-sample tracts. Exposure and covariates are population-weighted averages of tract values; models adjust for PM2.5, ozone, poverty, and the percentages of residents younger than 10 and 65 years or older (count models). IQR of ZIP-level park distance: 0.292 miles. Residual Moran's I of the negative binomial model without the spatial effect: 0.33 (p < 0.001). The weighted least-squares model uses the mean of the published age-adjusted rates, weighted by population, with HC1 standard errors. CI: confidence interval; IQR: interquartile range; PM2.5: fine particulate matter; HC1: heteroskedasticity-consistent standard errors with small-sample correction; ZIP: Zone Improvement Plan.

Model	Measure	Estimate	95% CI	p-value
Negative binomial, counts with person-years offset	Rate ratio per IQR	1.09	1.03 to 1.16	0.006
Negative binomial with spatial (Markov random field) effect	Rate ratio per IQR	0.99	0.93 to 1.04	0.631
Weighted least squares, age-adjusted rate	Rate difference per 10,000 per IQR	4.18	0.70 to 7.65	0.019

## Discussion

In this county-wide ecological analysis of 2,305 Los Angeles County census tracts, street-network park distance was not consistently associated with either outcome. In conventional population-weighted models adjusted for PM2.5, ozone, and poverty, tracts farther from parks had slightly higher AMI ED visit rates. The asthma estimate was also positive, but it was not statistically significant. Park distance accounted for less than 1% of the residual variation, and the straight-line measure from the tract centroid gave larger estimates. However, neither association persisted in spatial error models. The positive coefficients arose from differences between ZIP codes and broader regions of the county, as opposed to differences between neighboring tracts, although the data carry little information on the latter. Poverty was positively associated with both outcomes in the conventional and spatial models, and with asthma in the ZIP-code count models.

Earlier Los Angeles studies have associated public parks and open space [5] and urban tree cover [25] with lower asthma ED utilization. The present results are only weakly compatible with those findings at the county scale. Park-poor parts of the county tended to have higher ED visit rates in conventional adjusted models (statistically significant for AMI but not for asthma). However, the bivariate association between street-network park distance and either outcome was essentially null, and the positive adjusted coefficients appeared only after adjustment for poverty, PM2.5, and ozone. Each of these covariates is correlated with park distance across the county (tracts farther from parks had lower poverty, lower PM2.5, and higher ozone).

The contrast between the conventional and spatial error models is consistent with spatial confounding. When an exposure shares a broad geographic gradient with unmeasured influences on the outcome, ordinary regression can attribute part of that gradient to the exposure. Adding spatially correlated errors can change the exposure coefficient substantially, but that change is not a reliable adjustment for the unmeasured influences and can also distort a genuine association [26]. Furthermore, the structure of the outcome data makes this question hard to resolve. The outcome rates were calculated for ZIP codes and then distributed to tracts [22], so little of their variation (under a fifth) lies within ZIP codes, and within-ZIP differences in the outcomes largely reflect how ZIP-code rates were apportioned. Thus, a near-zero within-ZIP coefficient is not evidence of no local association.

Spatial lag and spatial Durbin models, which are unweighted, gave no statistically significant park-distance estimates, direct or indirect, for either outcome (Appendix 2). The spatial specifications therefore do not support a local association: the coefficients were negative in the error models and not statistically significant in the lag and Durbin models. At the same time, positive associations without a spatial term also appeared in unsmoothed ZIP-code counts, so they are not solely a product of the smoothing. Taken together, the most reasonable interpretation has two parts. First, after accounting for poverty and the two pollutants, tracts farther from parks had higher AMI ED visit rates and higher asthma ED visit rates between ZIP codes and regions, as a feature of the county's regional geography. Second, these data cannot divide that pattern into local park-related differences and regional confounding.

The negative park-distance coefficients in the spatial error models should not be interpreted as evidence that living farther from parks is protective. The asthma coefficient was largely attributable to the Antelope Valley, and neither coefficient was statistically significant once that region was excluded. Furthermore, across the park-area and walkshed measures, the spatial coefficients pointed in inconsistent directions, although park acreage within 0.5 or 1 mile was the one access measure whose association with AMI kept its sign in the spatial model. Finally, the conventional association was weaker with the street-network measure than with the straight-line measure from the tract centroid, so the imprecision of the simpler measure was not masking a larger association.

The same considerations apply to the pollutant coefficients. PM2.5 and ozone varied almost entirely between ZIP codes (97% and 99% of their variance), and park distance was correlated with both (tracts farther from parks had lower PM2.5 and higher ozone). In the region, park-adjacent neighborhoods have been found to have some of the highest nitrogen dioxide and PM2.5 concentrations [15], and traffic is the dominant contributor to PM2.5 organic carbon across the basin [27]. Additionally, in parks in four Los Angeles communities, visitors' ozone dose was higher in inland than in coastal communities of similar income [28]. Thus, the negative adjusted coefficients for PM2.5 and ozone in the conventional models are more consistent with regional confounding than with any protective association. Within ZIP codes, PM2.5 was positively associated with asthma rates, and in the spatial error models, the PM2.5 coefficients were positive. Ozone varies along the county's coastal-to-inland gradient and is lowest in the dense urban core, where ED visit rates are highest. In the spatial error models, its coefficients remained negative but were not statistically significant. Because nearly all of its variation is regional, its coefficient cannot be separated from that regional pattern either. These results do not call the documented harms of both pollutants [6-8,12] into question.

Poverty was associated with both outcomes in all the main models, consistent with the established relationship between socioeconomic status and cardiovascular outcomes [13] and with the social patterning of asthma morbidity and emergency care reliance [4]. Poverty's association with AMI rates is also in line with evidence that cardiovascular disease is shaped by cumulative socioeconomic conditions across the life course [29]. Additionally, ED visit rates measure utilization, not disease prevalence alone: communities with limited access to preventive and primary care rely more heavily on emergency departments [4], so part of the geographic pattern may reflect differences in healthcare access. Because parks vary widely in the facilities and activities they support [30], distance alone is a limited measure of access. The study covers a single county whose outcome data are modeled from ZIP-code records, so these patterns should be examined in other settings rather than assumed to generalize.

Limitations

This study has several limitations. First, it is a cross-sectional ecological study: tract-level associations cannot be applied to individuals (the ecological fallacy), no causal or temporal inference is possible, and results may depend on the areal unit (the modifiable areal unit problem). Second, the outcomes are spatially modeled ZIP-code rates distributed to tracts, so the unit of analysis is finer than the unit at which the outcome was generated. The modeling of rates for ZIP codes with fewer than 12 visits also pulls extreme rates toward local and statewide averages. The check against unsmoothed counts was possible only for asthma, because no comparable public ZIP-code counts were identified for AMI. The residuals of the conventional models were strongly spatially autocorrelated, and the conventional and spatial models gave different answers. Additionally, neither model isolates a local association. Third, the exposure is a distance along road centerlines from the population-weighted center of each tract, and it ignores park use and park quality, size, and amenities. A single point per tract also cannot represent how residents are spread within large tracts. The street network is an approximation, since it uses road centerlines instead of a pedestrian network and treats every road other than limited-access highways and ramps as walkable (including private roads and vehicular trails), while containing only the few pedestrian ways present in the TIGER/Line file. It reflects physical barriers only where they interrupt the road network. Furthermore, it matches 2016 roads to a 2024 park layer and 2010 population centers, and it joins each tract center and park boundary to the nearest road by a straight line. The park layer also includes restricted-access features and overlapping polygons. Restricting it to open-access features or merging the overlaps gave weaker conventional associations for asthma (1.57 and 0.85 per IQR) and, for AMI, a slightly larger estimate with open-access features (0.41) and a smaller one with the overlaps merged (0.23). The straight-line measure from the tract centroid gave larger conventional estimates, and the single nearest park, park acreage, and a 2016 pedestrian-network walkshed gave results that were consistent in direction in conventional models but inconsistent in spatial error models. Thus, the conclusions are limited more by the resolution of the outcome data than by the choice of access measure. Furthermore, none of these measures captures actual park use. Fourth, the park inventory (2024, compiled from 2023 sources) postdates the outcomes (2015-2017) and is only a lagged proxy for park access during the outcome period, and ozone (2017-2019) only partly overlaps it. Parks created after 2017 would make some tracts appear closer to parks than they were during the outcome period. The assumption that such error would weaken rather than create an association is supported by the county's 2016 inventory: when parks were taken from that inventory, so that parks, roads, and outcomes share one period, the street-network measure correlated 0.97 with the primary exposure and gave similar estimates (1.84, 95% CI 0.02 to 3.66, and 0.37 per IQR, compared with 1.64 and 0.34). With 2012-2014 ozone, alone or averaged with the 2017-2019 values, the asthma estimates were 1.25 and 1.51. Fifth, the magnitude of the conventional association depended on how large peripheral tracts were handled: it was larger when tracts beyond 1.25 or 1.0 mile by the straight-line measure were excluded, and these thresholds were chosen after inspecting that measure's distribution. Sixth, as discussed above, the negative pollutant coefficients in the conventional models indicate that these models are misspecified. The coefficients are better explained by regional confounding than by protection, and they show that these models do not isolate the contribution of any single factor. Seventh, residual confounding is likely, since poverty reflects, but does not fully capture, correlated factors such as race and ethnicity, educational attainment, housing quality, healthcare access, and unemployment. The percentage of uninsured residents was the only one of these factors examined, and it entered an additional model rather than the main ones (Appendix 2). Eighth, most tracts lie within a narrow band of park distance (IQR 0.38 miles), so the main estimates are reported per IQR rather than per mile. Ninth, CalEnviroScreen reports no poverty estimate for 38 tracts and no ED visit rates for nine; these values had been recorded as zeros in the exported analysis file and were excluded here. Tenth, the park layer and the road network are clipped at the county boundary, so distances for border tracts may be overstated. Eleventh, the primary specification (the sample, population weighting, and the street-network exposure) was changed after the initial analysis, and the study was not preregistered. Models with the original specification (the straight-line measure without population weights) are reported among the sensitivity analyses (Tables 6, 7). Finally, the numerous models were not adjusted for multiple comparisons. One of several interaction terms (park distance with poverty, for asthma) was nominally statistically significant in a non-spatial model and is not interpreted.

## Conclusions

Across Los Angeles County, census tracts farther from parks had slightly higher AMI ED visit rates in conventional models adjusted for PM2.5, ozone, and poverty. The asthma estimate was also positive, but with the primary street-network measure it was not statistically significant and was smaller than with the straight-line distance. Both associations were small, appeared between ZIP codes and regions rather than within them, varied with the handling of large peripheral tracts, and did not persist in spatial error models. At the same time, poverty was the prespecified covariate most consistently associated with both outcomes.

Taken together, these findings suggest that the adjusted association between park distance and ED visit rates is best understood as one feature of the county's regional geography, rather than as evidence that park access itself explains where asthma and AMI ED visits concentrate. These tract-level data, whose outcomes are spatially modeled from ZIP-code records, cannot distinguish local park-related differences from regional confounding, and they do not support causal or prescriptive conclusions about park access and ED use. Outcome data measured at finer resolution, or studies of changes in park access over time, would be needed to determine whether park access itself is related to ED use at the neighborhood level.

## Figures and Tables

**Figure 6:**
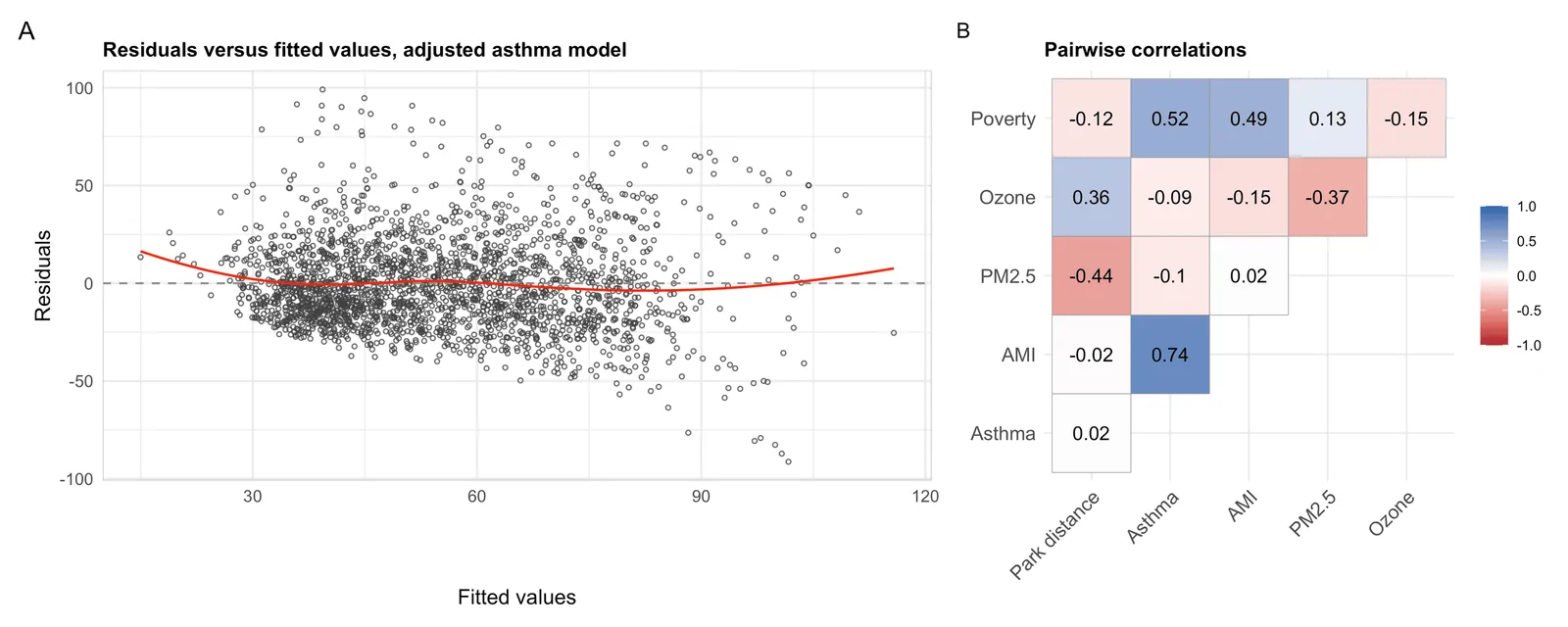
Model diagnostics (A) Residuals versus fitted values for the population-weighted adjusted asthma model in the primary sample (n = 2,305); the red line is a locally weighted smoother. (B) Pairwise correlations in the primary sample among park distance (mean street-network distance from the population-weighted tract center to the three nearest parks), the two outcomes (asthma and AMI ED visit rates), and the three covariates; panel labels are abbreviated to the variable name. AMI: acute myocardial infarction; ED: emergency department; PM2.5: fine particulate matter; Poverty: percentage of residents below twice the federal poverty level.

**Figure 7:**
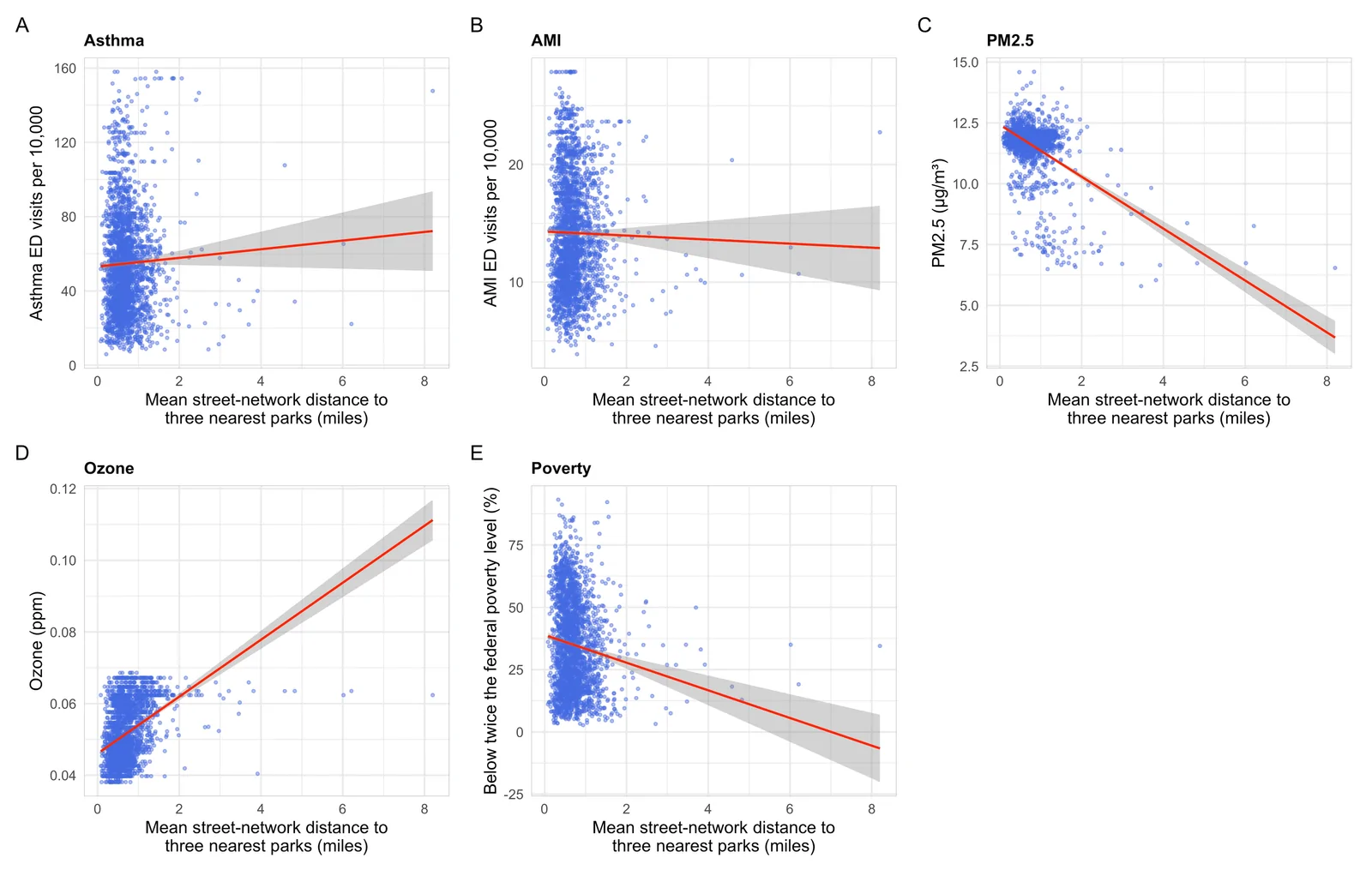
Bivariate scatterplots of street-network park distance and each study variable Park distance is the mean street-network distance from the population-weighted tract center to the three nearest parks. Each red line is the population-weighted least-squares fit with its 95% confidence band, primary sample (n = 2,305); the bands use conventional standard errors and are narrower than the ZIP-clustered intervals in Table 3. (A) Asthma ED visit rates per 10,000 residents. (B) AMI ED visit rates per 10,000 residents. (C) PM2.5. (D) Ozone. (E) Percentage of residents below twice the federal poverty level. A few tracts beyond three miles lie at the right of each panel. AMI: acute myocardial infarction; ED: emergency department; PM2.5: fine particulate matter; ppm: parts per million.

## Appendices

Appendix 1

Model diagnostics for the primary sample and bivariate scatterplots are shown below.

Appendix 2

The results of additional models are presented in the table below. The specification log deposited with the data and code lists the models estimated by the analysis code. In spatial lag models, the park distance was not statistically significantly associated with either outcome. In spatial Durbin models, which separate a tract's own park distance from that of its neighbors, neither the tract's own distance nor the distances in neighboring tracts were statistically significantly associated with either outcome (combined direct plus indirect estimate: asthma 4.58 per mile, 95% CI -7.71 to 16.86, p = 0.458, or 1.72 per IQR; AMI 1.13, 95% CI -0.74 to 2.99, p = 0.227, or 0.42 per IQR). The interaction of park distance with poverty was nominally statistically significant for asthma (0.36 per mile per percentage point, 95% CI 0.01 to 0.71, p = 0.045) but not for AMI, and the interactions with PM2.5 were not statistically significant. The interaction models are non-spatial and post hoc, and the term is not interpreted. Adding population density, alone or together with the percentage of uninsured residents, left the non-spatial park-distance coefficient not statistically significant for asthma (3.70 and 3.46 per mile) and close to its primary value for AMI (0.83 per mile, p = 0.028, and 0.76, p = 0.047). In models with three-digit ZIP-code fixed effects, park distance was not statistically significantly associated with either outcome.

**Table 9:** Other models estimated Exploratory models in the primary sample (n = 2,305; n = 2,304 when % uninsured is included). Park-distance terms are per mile (multiply by 0.376 for the estimate per IQR of park distance). Spatial lag and Durbin models are unweighted (the software does not support weights); their CIs use simulated standard errors and their p-values are simulation-based (1,000 simulations). All other models are population-weighted with standard errors clustered by ZIP code. The deposited specification log lists the models estimated by the analysis code. AMI: acute myocardial infarction; PM2.5: fine particulate matter; CI: confidence interval; IQR: interquartile range; ZIP: Zone Improvement Plan.

Model	Outcome	Term	Estimate	95% CI	p-value
Spatial lag model (unweighted)	Asthma	Own-tract (direct) estimate	-0.653	-2.312 to 1.006	0.414
Spatial lag model (unweighted)	Asthma	Neighbors' (indirect) estimate	-2.720	-9.684 to 4.243	0.417
Spatial lag model (unweighted)	Asthma	Total (direct plus indirect) estimate	-3.374	-11.991 to 5.244	0.416
Spatial lag model (unweighted)	AMI	Own-tract (direct) estimate	-0.026	-0.356 to 0.303	0.892
Spatial lag model (unweighted)	AMI	Neighbors' (indirect) estimate	-0.078	-1.059 to 0.903	0.891
Spatial lag model (unweighted)	AMI	Total (direct plus indirect) estimate	-0.104	-1.413 to 1.206	0.891
Spatial Durbin model (unweighted)	Asthma	Own-tract (direct) estimate	-0.631	-2.289 to 1.027	0.452
Spatial Durbin model (unweighted)	Asthma	Neighbors' (indirect) estimate	5.207	-6.190 to 16.603	0.363
Spatial Durbin model (unweighted)	Asthma	Total (direct plus indirect) estimate	4.576	-7.710 to 16.862	0.458
Spatial Durbin model (unweighted)	AMI	Own-tract (direct) estimate	-0.036	-0.340 to 0.268	0.827
Spatial Durbin model (unweighted)	AMI	Neighbors' (indirect) estimate	1.161	-0.558 to 2.881	0.178
Spatial Durbin model (unweighted)	AMI	Total (direct plus indirect) estimate	1.125	-0.737 to 2.988	0.227
Interaction	Asthma	Park distance × poverty	0.360	0.008 to 0.713	0.045
Interaction	Asthma	Park distance × PM2.5	0.058	-2.385 to 2.501	0.963
Interaction	AMI	Park distance × poverty	0.023	-0.028 to 0.074	0.375
Interaction	AMI	Park distance × PM2.5	0.074	-0.260 to 0.407	0.664
Adjusted additionally for population density	Asthma	Park distance	3.702	-0.914 to 8.318	0.116
Adjusted additionally for population density	AMI	Park distance	0.832	0.089 to 1.576	0.028
Adjusted additionally for density and % uninsured	Asthma	Park distance	3.463	-1.250 to 8.176	0.149
Adjusted additionally for density and % uninsured	AMI	Park distance	0.758	0.009 to 1.508	0.047
Three-digit ZIP-code fixed effects	Asthma	Park distance	-1.797	-5.854 to 2.259	0.384
Three-digit ZIP-code fixed effects	AMI	Park distance	0.151	-0.421 to 0.723	0.603

## References

[1] Wallace DD, Han B, Cohen DA, Derose KP (2022). The effects of park-based interventions on health-related outcomes among youth: a systematic review. Am J Health Promot.

[2] Connolly R, Lipsitt J, Aboelata M, Yañez E, Bains J, Jerrett M (2023). The association of green space, tree canopy and parks with life expectancy in neighborhoods of Los Angeles. Environ Int.

[3] Clark NA, Demers PA, Karr CJ, Koehoorn M, Lencar C, Tamburic L, Brauer M (2010). Effect of early life exposure to air pollution on development of childhood asthma. Environ Health Perspect.

[4] Grant T, Croce E, Matsui EC (2022). Asthma and the social determinants of health. Ann Allergy Asthma Immunol.

[5] Douglas JA, Archer RS, Alexander SE (2019). Ecological determinants of respiratory health: Examining associations between asthma emergency department visits, diesel particulate matter, and public parks and open space in Los Angeles, California. Prev Med Rep.

[6] Zhang Y, Yin X, Zheng X (2023). The relationship between PM2.5 and the onset and exacerbation of childhood asthma: a short communication. Front Pediatr.

[7] Grzywa-Celińska A, Krusiński A, Milanowski J (2020). 'Smoging kills' - effects of air pollution on human respiratory system. Ann Agric Environ Med.

[8] Krittanawong C, Qadeer YK, Hayes RB, Wang Z, Thurston GD, Virani S, Lavie CJ (2023). PM(2.5) and cardiovascular diseases: state-of-the-art review. Int J Cardiol Cardiovasc Risk Prev.

[9] Srebot V, Gianicolo EA, Rainaldi G, Trivella MG, Sicari R (2009). Ozone and cardiovascular injury. Cardiovasc Ultrasound.

[10] Juda-Rezler K, Reizer M, Maciejewska K, Błaszczak B, Klejnowski K (2020). Characterization of atmospheric PM(2.5) sources at a Central European urban background site. Sci Total Environ.

[11] Chauhan BVS, Berg MJ, Sharma A, Smallbone KL, Wyche KP (2025). Temporal and machine learning-based principal component and clustering analysis of VOCs and their role in urban air pollution and ozone formation. Atmosphere (Basel).

[12] Kim SY, Kim E, Kim WJ (2020). Health effects of ozone on respiratory diseases. Tuberc Respir Dis (Seoul).

[13] Schultz WM, Kelli HM, Lisko JC (2018). Socioeconomic status and cardiovascular outcomes: challenges and interventions. Circulation.

[14] Kim D, Ahn Y (2021). The contribution of neighborhood tree and greenspace to asthma emergency room visits: an application of advanced spatial data in Los Angeles County. Int J Environ Res Public Health.

[15] Su JG, Jerrett M, de Nazelle A, Wolch J (2011). Does exposure to air pollution in urban parks have socioeconomic, racial or ethnic gradients?. Environ Res.

[16] Jiang Y, Yang Y (2022). Environmental justice in Greater Los Angeles: impacts of spatial and ethnic factors on residents' socioeconomic and health status. Int J Environ Res Public Health.

[17] von Elm E, Altman DG, Egger M, Pocock SJ, Gøtzsche PC, Vandenbroucke JP (2007). The Strengthening the Reporting of Observational Studies in Epidemiology (STROBE) statement: guidelines for reporting observational studies. Lancet.

[18] Magill PL, Benoliel RW (1952). Air pollution in Los Angeles County. Ind Eng Chem.

[19] Zhao Y, Li Y, Li Y, Kumar A, Kleeman MJ (2025). Southern California ozone exposure disparities under different emissions control strategies in a low-carbon future. Sci Total Environ.

[20] Krieger N (2006). A century of census tracts: health & the body politic (1906-2006). J Urban Health.

[21] (2026). Los Angeles County Department of Public Health: Recreational spaces (Community Health Profiles Data Initiative). http://lacounty.hub.arcgis.com/datasets/4e6083c7960d4a1caedb57aa76473e4f.

[22] August L, Bangia K, Plummer L, Prasad S, Ranjbar K, Slocombe A, Wieland W (2026). CalEnviroScreen 4.0 report. https://oehha.ca.gov/media/downloads/calenviroscreen/report/calenviroscreen40reportf2021.pdf.

[23] (2026). OpenStreetMap Foundation: Copyright and license. https://www.openstreetmap.org/copyright.

[24] Faust J, August L, Bangia K (2026). Update to the California Communities Environmental Health Screening Tool: CalEnviroScreen 3.0. https://data.ca.gov/dataset/0bd5f40b-c59b-4183-be22-d057eae8383c/resource/850cbbb8-f616-48c0-9240-971003c99413/download/calenviroscreen-3.0-report.pdf.

[25] Chen P, Hanlon B (2025). Urban green space, respiratory health and rising temperatures: an examination of the complex relationship between green space and adult asthma across racialized neighborhoods in Los Angeles County. Landsc Urban Plan.

[26] Hodges JS, Reich BJ (2010). Adding spatially-correlated errors can mess up the fixed effect you love. Am Stat.

[27] Soleimanian E, Mousavi A, Taghvaee S, Sowlat MH, Hasheminassab S, Polidori A, Sioutas C (2019). Spatial trends and sources of PM2.5 organic carbon volatility fractions (OCx) across the Los Angeles Basin. Atmos Environ.

[28] Winter PL, Padgett PE, Milburn LS, Li W (2019). Neighborhood parks and recreationists' exposure to ozone: a comparison of disadvantaged and affluent communities in Los Angeles, California. Environ Manage.

[29] Pollitt RA, Rose KM, Kaufman JS (2005). Evaluating the evidence for models of life course socioeconomic factors and cardiovascular outcomes: a systematic review. BMC Public Health.

[30] Dietz LW, Šćepanović S, Zhou K, Zanella AF, Quercia D (2025). Understanding the potential of urban parks to promote well-being. Nat Cities.

